# The budding yeast Start repressor Whi7 differs in regulation from Whi5, emerging as a major cell cycle brake in response to stress

**DOI:** 10.1242/jcs.251413

**Published:** 2020-12-21

**Authors:** Ester Méndez, Mercè Gomar-Alba, M. Carmen Bañó, Manuel Mendoza, Inma Quilis, J. Carlos Igual

**Affiliations:** 1 Estructura de Recerca Interdisciplinar en Biotecnologia i Biomedicina (ERI BIOTECMED) and Departament de Bioquímica i Biologia Molecular, Universitat de València, 46100 Burjassot (Valencia), Spain; 2 Institut de Génétique et de Biologie Moléculaire et Cellulaire, 67404 Illkirch, France; 3 Centre National de la Recherche Scientifique, UMR7104, 67404 Illkirch, France; 4 Institut National de la Santé et de la Recherche Médicale, U964, 67404 Illkirch, France; 5 Université de Strasbourg, 67000 Strasbourg, France

**Keywords:** Cell cycle, Start, Whi7, Whi5, Stress

## Abstract

Start is the main decision point in the eukaryotic cell cycle at which cells commit to a new round of cell division. It involves the irreversible activation of a transcriptional programme through the inactivation of Start transcriptional repressors: the retinoblastoma family in mammals, or Whi5 and its recently identified paralogue Whi7 (also known as Srl3) in budding yeast. Here, we provide a comprehensive comparison of Whi5 and Whi7 that reveals significant qualitative differences. Indeed, the expression, subcellular localization and functionality of Whi7 and Whi5 are differentially regulated. Importantly, Whi7 shows specific properties in its association with promoters not shared by Whi5, and for the first time, we demonstrate that Whi7, and not Whi5, can be the main contributor to Start inhibition such as it occurs in the response to cell wall stress. Our results help to improve understanding of the interplay between multiple differentially regulated Start repressors in order to face specific cellular conditions.

## Abstract (precis)

**Highlighted Article:** Cells can use the interplay between functionally redundant but differentially regulated cell-cycle repressors in order to confer new repression capabilities and to respond to specific cellular conditions.

## INTRODUCTION

Eukaryotic cell division is controlled by a complex network of intertwining regulatory circuits (Morgan, 2007). At the heart of this system is the conserved cyclin dependent kinase (CDK) family (Malumbres, 2014). Distinct CDK complexes are activated in a cell cycle phase-specific order, which allows the phosphorylation of many other proteins that drive cell cycle progression. A hallmark of the cell cycle regulatory system is its extraordinary robustness. This is achieved by the existence of different regulatory mechanisms that affect a particular process, and functionally redundant proteins able to substitute for each other or act in different cellular contexts.

The G1-to-S phase transition (Start) represents a critical point at which cells irreversibly commit to initiate a new cell cycle. The Start regulatory network is well conserved between yeast and mammalian cells (Johnson and Skotheim, 2013). The activation of G1 CDKs initiates a wave of gene expression through the inhibition of specific transcriptional repressors, which provides key cell cycle regulators and effectors of downstream cell cycle events. Positive and negative feedback loops guarantee the coherent activation and inactivation of this transcriptional programme (Bertoli et al., 2013). In the yeast *Saccharomyces cerevisiae*, activation of Start gene expression is controlled by the CDK Cdc28 associated with the G1 cyclins Cln1, Cln2 and Cln3, and involves the transcription factors SBF (Swi4–Swi6) and MBF (Mbp1–Swi6) (Haase and Wittenberg, 2014). SBF associates with its target promoters in early G1, but its activity is blocked due to the binding of the transcriptional repressor Whi5 (Costanzo et al., 2004; de Bruin et al., 2004), which mediates the recruitment of histone deacetylase activities to promoters (Huang et al., 2009; Takahata et al., 2009; Wang et al., 2009). Expression is initially triggered by the Cdc28–Cln3 kinase in late G1, when it extensively phosphorylates the Whi5 repressor and the SBF and MBF transcriptional factors, activating gene expression (Costanzo et al., 2004; de Bruin et al., 2004; Palumbo et al., 2016; Wagner et al., 2009). This process is coordinated with cell growth, although the molecular mechanism is still a matter of controversy (Aldea et al., 2017; Dorsey et al., 2018; Heldt et al., 2018; Litsios et al., 2019; Moreno et al., 2019; Schmoller et al., 2015; Wang et al., 2009). As a result of this initial activation, Cdc28–Cln1 and Cdc28–Cln2 accumulate and further phosphorylate Whi5 and Swi6, promoting the complete dissociation of the Whi5 repressor. This positive-feedback mechanism strengthens a sharp transcriptional response and makes the Start transition coherent and irreversible (Charvin et al., 2010; Skotheim et al., 2008). Later in the cell cycle, SBF-dependent transcription is silenced by Cdc28–Clb CDKs (Koch et al., 1996), whereas MBF is inactivated in a negative feedback loop by the transcriptional repressor Nrm1, whose gene is regulated by MBF (de Bruin et al., 2006). A similar regulatory circuitry controls Start (referred to as the restriction point) in mammalian cells, where the roles of Cdc28–Cln3, SBF/MBF, Whi5 and Cdc28–Cln1/Cln2 are played by CDK4/6–cyclin D, the E2F–DP transcription factor family, retinoblastoma transcriptional repressors [Rb (also known as RB1), p107 (RBL1) and p130 (RBL2)] and CDK2–cyclin E, respectively (Bertoli et al., 2013; Johnson and Skotheim, 2013).

Although SBF and MBF preferentially regulate a subset of specific genes (cyclins *CLN1* and *CLN2*, and cell wall genes for SBF; cyclins *CLB5* and *CLB6*, and DNA metabolism genes for MBF), there is an important functional redundancy between them: SBF can bind to the MBF binding sites and vice versa, one factor can replace the other in its absence for a significant number of genes, and there are common target genes for SBF and MBF (Bean et al., 2005; Ferrezuelo et al., 2010). In fact, in some genes there is an SBF-to-MBF switch during the G1-S transition (Bastos de Oliveira et al., 2012). The crosstalk between both transcription factors also includes the regulation of the *SWI4* gene by MBF (Harris et al., 2013). The functional redundancy between SBF and MBF, as well as the importance of the Start transcriptional programme, are evidenced by the lethality of the *swi4 swi6* and *swi4 mbp1* double mutations (Koch et al., 1993). Strikingly, *mbp1 swi6* cells are viable, which indicates that Swi4 could act alone in the absence of Swi6.

An important aspect of cellular behaviour is the ability to properly respond to changes in environmental conditions. To overcome these stresses, cells process and integrate information from their environment into the cell cycle control network, in particular at Start (Aldea et al., 2017; Ewald, 2018). In fact, multiple signalling pathways impinge on different regulators to promote or prevent passage through Start: molecular mechanisms connect the response to nutrient availability with Swi4 (Amigoni et al., 2015), Whi5 (Talarek et al., 2017), the cyclin-dependent kinase inhibitor Sic1 (Moreno-Torres et al., 2015), the SBF-interacting proteins Msa1 and Msa2 (Miles et al., 2016) or the cyclin Cln3 (Gallego et al., 1997; Menoyo et al., 2013); osmotic stress targets Sic1 (Escote et al., 2004), Whi5 (Gonzalez-Novo et al., 2015) and Cip1 (Chang et al., 2017); and genotoxic stress affects transcriptional factors Nrm1 (Travesa et al., 2012), Swi6 (Sidorova and Breeden, 1997) as well as Cip1 (Zhang et al., 2017). Therefore, multiple regulatory pathways might cooperate in regulating the G1-S transition for cells to cope with environmental changes.

One of the *S. cerevisiae* signalling pathways involved in the response to environmental conditions is the CWI (cell wall integrity) or PKC (protein kinase C) pathway (Levin, 2011). This pathway responds to a variety of conditions that challenge the cells – such as thermal, osmolarity and pH changes, cell wall perturbing agents and oxidative stress – as well as to endogenous cellular processes such as polarized growth. The main function of the pathway is the maintenance of cell integrity in these particular conditions, which have an impact on the cell surface (cell wall and/or plasma membrane) integrity. This stress is detected by sensor proteins that activate the protein kinase C (Pkc1), which controls the activity of the MAPK Slt2 cascade, which in turn induces the expression of genes important for cell wall remodelling and morphogenesis in order to fortify the cell wall and maintain its integrity (Sanz et al., 2017). This transcriptional response is mostly executed by Rlm1, whose activity is regulated by Slt2-mediated phosphorylation. Slt2 also controls the expression of a small subset of cell wall genes through the regulation of the transcription factor SBF by a non-catalytic mechanism (Baetz et al., 2001; Kim et al., 2008; Truman et al., 2009). Importantly, the CWI pathway has a more general role, because components of the pathway also affect other aspects of yeast cell biology (Heinisch and Rodicio, 2018; Jiménez-Gutierrez et al., 2020).

Direct connections between components of the CWI pathway with the regulation of the cell cycle have been described. As regards the G1-S transition, Slt2 phosphorylates Swi6, blocking its nuclear import in response to cell wall stress (Kim et al., 2010), and Sic1, restraining the progression to S phase (Moreno-Torres et al., 2015, 2017), and Rlm1 delays Start in cells grown on a poor medium (Piccirillo et al., 2017). Additionally, the CWI pathway acts on different targets to negatively (Darieva et al., 2012; Harrison et al., 2001; Negishi et al., 2016; Yano et al., 2013) or positively (Thai et al., 2017) regulate the G2-M transition. All these data highlight the central role of the CWI pathway in the response to a wide variety of environmental challenges. However, we still lack a complete picture of how the pathway affects the cell cycle regulatory system to coordinate cell proliferation with the response to such a diverse set of environmental conditions.

Whi7 (also known as Srl3), Whi5 and Nrm1 constitute a family of proteins characterized by the presence of a GTB (G1/S transcription binding factor) motif (Travesa et al., 2013). *WHI7* gene expression is induced in different situations of cellular stress (Garcia et al., 2004; Gasch et al., 2000; Waern and Snyder, 2013), and Whi7 protein is unstable, being degraded by the SCF^Grr1^ ubiquitin ligase in a Cdc28-dependent and cell cycle-regulated way (Gomar-Alba et al., 2017). Unlike *whi5* mutation, *whi7* mutation has no effect on cell size distribution in exponentially growing cultures, questioning a role for this protein at the beginning of the cell cycle (Costanzo et al., 2004). However, Whi7 acts as a negative regulator of Start, helping to retain the Cdc28–Cln3 complex at the ER surface (Yahya et al., 2014), and loss of Whi7 has a slight effect on re-entry into the cell cycle after quiescence (Miles et al., 2016). More recently, our group reported that Whi7 acts as a transcriptional repressor of SBF-dependent transcription, acting as a genuine Whi5 paralogue (Gomar-Alba et al., 2017). However, little is known about the mechanisms of action of Whi7 and how they potentially differ from those of Whi5. The different effects of *whi5* and *whi7* mutations on cell size indicate that Whi5 has a greater relevance than Whi7 during the Start transition. The differences between cellular levels of Whi5 and Whi7, caused by the high instability of Whi7 protein (Gomar-Alba et al., 2017), could contribute to explaining this; however, we wonder whether, in addition to this quantitative difference, Whi7 and Whi5 could also show qualitative differences. In this work, we have carried out a comprehensive comparison of Whi7 and Whi5 Start repressors. The results reveal significant intrinsic differences in the regulation and function of both proteins and a major role for Whi7 in Start control during the response to cell wall stress.

## RESULTS

### Comparative analysis of Whi7 and Whi5 association with promoters of target genes

**Fig. 1.**
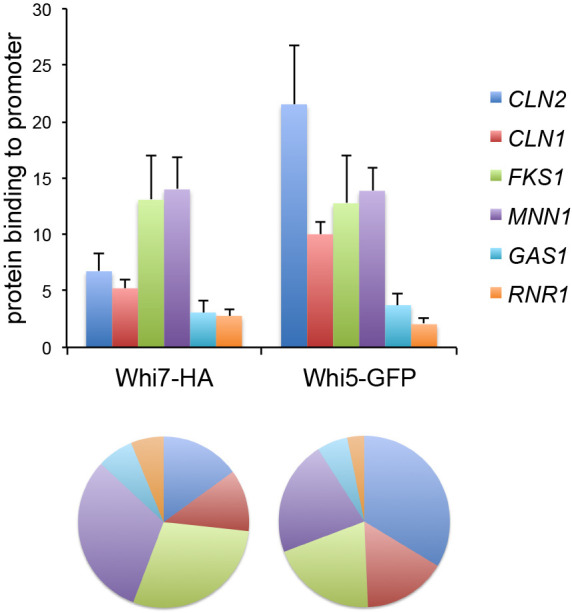
**Whi7 and Whi5 show different binding preferences for Start gene promoters.** Whi7 binding to *CLN2*, *CLN1*, *FKS1*, *MNN1*, *GAS1* and *RNR1* promoters was investigated using ChIP assays in *WHI5–GFP* (JCY2116) cells transformed with the centromeric pWHI7–HA plasmid. The wild-type W303-1a strain transformed with an empty vector was used as a no-tag control in the assays. Top: graph represents Whi7 and Whi5 binding relative to the no-tag control for each gene. Data are presented as mean±s.d. of three experiments. Bottom: a chart with the relative binding pattern of the analysed genes is shown.

We first wondered whether Whi7 and Whi5 could show qualitative differences in their affinity for target genes. The Whi7 and Whi5 repressors are associated with the transcription factor SBF to bind to Start gene promoters (Costanzo et al., 2004; de Bruin et al., 2004; Gomar-Alba et al., 2017). Because this binding is strongly periodic and occurs only at a very specific moment in the cell cycle, we decided to analyse the association of both Whi7 and Whi5 with promoters in the same cells to accurately investigate differences in their binding. The genes analysed included the G1/S cyclin genes *CLN2* and *CLN1*, and genes encoding proteins involved in cell wall and DNA biosynthesis, such as the glucan synthase Fks1, the mannosyltransferase Mnn1, the glucanosyltransferase Gas1 and the ribonucleotide reductase subunit Rnr1. The *CLN2*, *CLN1*, *FKS1*, *MNN1* and *GAS1* genes are mainly regulated by SBF, whereas regulation of the *RNR1* gene depends mainly on MBF (Harris et al., 2013). The results show that both Whi7 and Whi5 were able to bind to the promoters of all genes. However, their relative binding pattern to these genes was different, revealing that Whi5 had a marked preference for the Start cyclin genes (in particular *CLN2*), and Whi7 for the cell wall genes (*FKS1* and *MNN1*) (Fig. 1). This suggests that Whi7 and Whi5 association with promoters involves some distinct mechanisms.

### Differential determinants in the binding of Whi7 and Whi5 to promoters

**Fig. 2.**
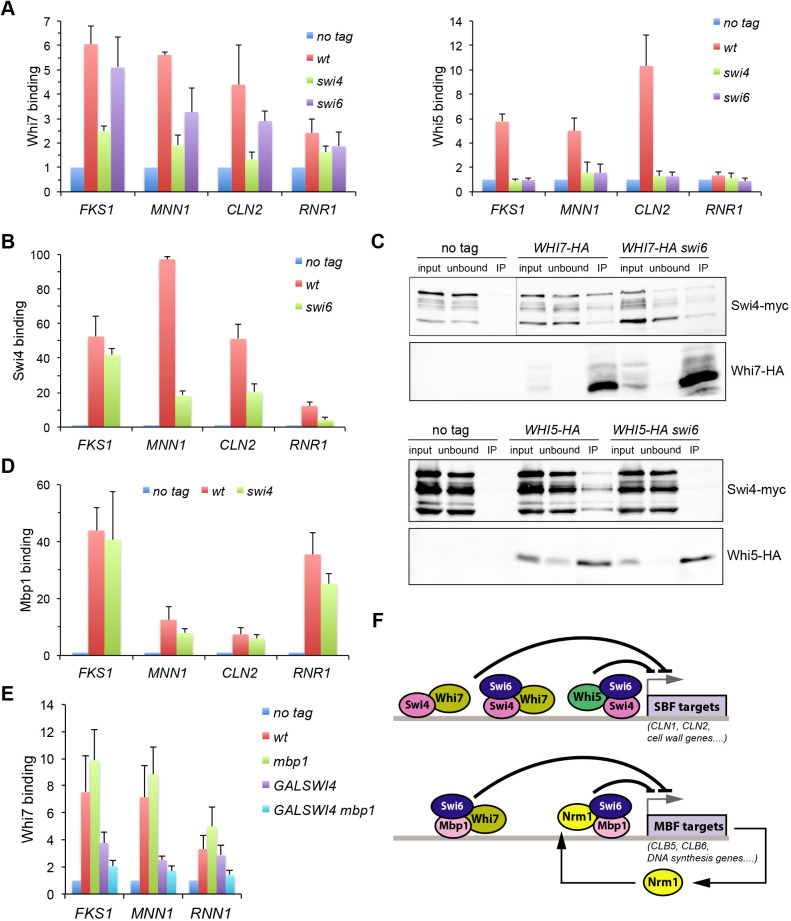
**Whi7 and Whi5 present differences in the Start transcription factors required for binding to promoters.** (A) Left: Whi7 binding to *FKS1*, *MNN1*, *CLN2* and *RNR1* promoters was investigated using ChIP assays in wild-type (wt; W303-1a), *swi4* (JCY167) and *swi6* (JCY325) cells transformed with centromeric pWHI7–HA plasmid. W303-1a transformed with an empty vector was used as the no-tag control (no tag). Right: Whi5 binding to the same promoters was investigated using ChIP assays in cultures of the W303-1a (no tag), *WHI5–HA* (wt; JCY1346), *WHI5–HA swi4* (JCY740) and *WHI5–HA swi6* (JCY1934) strains. Graphs represent Whi7 and Whi5 binding relative to the no-tag control for each gene. Data are presented as mean±s.d. of at least three experiments. (B) Swi4 binding to *CLN2*, *RNR1, FKS1* and *MNN1* promoters was investigated using ChIP assays in cultures of the W303-1a (no tag), *SWI4–HA* (wt; JCY1956) and *SWI4–HA swi6* (JCY1958) strains. Graph represents Swi4 binding relative the no-tag control for each gene. Data are presented as mean±s.d. of three experiments. (C) Top: *SWI4–myc* (JCY1879) and *SWI4–myc swi6* (JCY1884) cells were transformed with the centromeric pWHI7–HA plasmid (*WHI7–HA* and *WHI7–HA swi6*, respectively), alongside *SWI4–myc* cells transformed with a control vector (no tag). Whi7 was immunoprecipitated from crude extracts, and the presence of Whi7 and Swi4–myc in the input, unbound and immunoprecipitated (IP) fractions was determined by western blotting. Bottom: Whi5 was immunoprecipitated from crude extracts from no-tag control *SWI4–myc* (no tag; JCY1879), *SWI4–myc WHI5–HA* (JCY2134) and *SWI4–myc WHI5–HA swi6* (JCY2135) cells, and the presence of Whi5 and Swi4–myc in the input, unbound and immunoprecipitated fractions was determined by western blotting. Input, 5%; unbound, 5%. (D) Mbp1 binding to *CLN2*, *RNR1, FKS1* and *MNN1* promoters was investigated using ChIP assays in cultures of the W303-1a (no tag), *MBP1–HA* (wt; JCY2084) and *MBP1–HA swi4* (JCY2100) strains. Graph represents Mbp1 binding relative to the no-tag control for each gene. Data are presented as mean±s.d. of three experiments. (E) Wild-type (wt; W303-1a), *mbp1* (JCY2194), *GAL1:SWI4* (JCY2268) and *mbp1 GAL1:SWI4* (JCY2304) cells transformed with the centromeric pWHI7–HA plasmid were grown in SGal and incubated overnight in SD medium to repress *SWI4* expression. Whi7 binding to *FKS1*, *MNN1* and *RNR1* promoters was investigated using ChIP assays. W303-1a transformed with an empty vector was used as the no-tag control. Graph represents Whi7 binding relative to the no-tag control for each gene. Data are presented as mean±s.d. of at least six experiments. (F) Scheme of Whi7 binding to gene promoters of the Start transcriptional programme (see Discussion).

Whi5 binding to SBF and Start gene promoters depends on the integrity of the transcription factor, because the absence of either of its components (Swi4 or Swi6) abolishes the interaction (Costanzo et al., 2004; de Bruin et al., 2004). We previously described that Whi7 binds to the *CLN2* promoter mainly through SBF (Gomar-Alba et al., 2017). To obtain further insight into the association between Whi7 and the transcription factor SBF, the binding of Whi7 to the Start genes *CLN2*, *FKS1*, *MNN1* and *RNR1* in *swi4* and *swi6* mutant strains was investigated. In agreement with the fact that Whi5 interaction with SBF requires the intact transcription factor complex, Whi5 binding to promoters was completely lost in the absence of either Swi4 or Swi6. Strikingly, this was not the case for Whi7. Whi7 was still substantially associated with all genes in the absence of Swi6, although to a lesser extent (Fig. 2A). We envisaged the possibility that Swi4 alone could mediate this binding. Supporting this, deletion of Swi6 reduced, but did not abolish, association of Swi4 with *CLN2*, *FKS1*, *MNN1* and *RNR1* promoters (Fig. 2B). Importantly, co-immunoprecipitation experiments demonstrated that unlike Whi5, Whi7 was able to physically interact *in vivo* with Swi4 in the absence of Swi6 (Fig. 2C). All these results demonstrate that, whereas Whi5 only interacts with the integral transcriptional factor SBF, Whi7 is also able to associate with Swi4 in the absence of Swi6 in order to bind to Start promoters. On the other hand, Whi7 association was drastically reduced in the absence of Swi4, especially in the case of the *CLN2* promoter (Fig. 2A). This result reflects that the Whi7 binding to promoters is strongly dependent on Swi4, either when it is part of the SBF complex with Swi6 or on its own. However, it is important to note that, unlike for Whi5, a residual binding of Whi7 was still observed in the absence of Swi4, mainly to the *FKS1* and *MNN1* promoters. We wondered whether this residual binding in the absence of Swi4 could be due to MBF. Supporting this, Mbp1 was found to be associated with all the analysed promoters in both a wild-type strain and the *swi4* mutant (Fig. 2D). More importantly, inactivation of Mbp1 in the absence of Swi4 caused a reduction in Whi7 binding to *FKS1*, *MNN1* and *RNR1* promoters (Fig. 2E). Although no physical interaction between Whi7 and Mbp1 could be detected by co-immunoprecipitation assays, this observation strongly suggests that in the absence of Swi4, MBF mediates Whi7 recruitment to target genes. In short, although both Whi5 and Whi7 share a function as Start transcriptional repressors, their association with target genes involves specific molecular determinants (Fig. 2F).

### Cell-cycle regulation of Whi7 subcellular localization

**Fig. 3.**
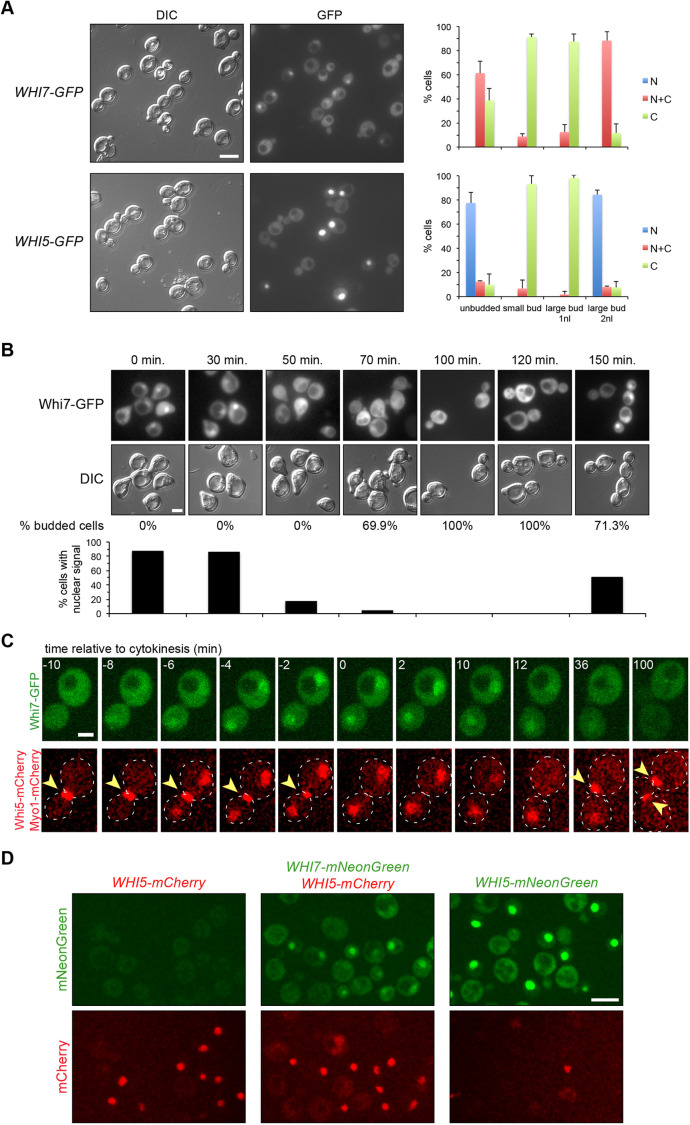
**Whi7 subcellular localization is cell cycle regulated.** (A) Exponentially growing cells of the wild-type (W303-1a) strain transformed with plasmids pADH1:WHI7–GFP_4_ or pADH1:WHI5–GFP_4_ were analysed by fluorescence microscopy. GFP signal and DIC images are shown. Cells were scored as unbudded, budded with one nucleus (1nl) or budded with two nuclei (2nl), and as cells with fluorescence signal only in the nuclei (N), in the nuclei and the cytoplasm (N+C) or only in the cytoplasm (C). Graphs show mean±s.d. percentage protein localization derived from three (Whi7) or two (Whi5) independent experiments (*n*>300). (B) Exponentially growing cells of the wild-type (W303-1a) strain transformed with plasmid pADH1:WHI7–GFP_4_ were blocked in G1 using α-factor. The localization of Whi7 was investigated at the indicated times after the release from the arrest. Budding index (% budded cells) is shown as an indication of cell cycle progression. Graph shows the percentage of cells in which nuclear signal was detected. One of two replicates is shown. (C) Time-lapse analysis by confocal fluorescence microscopy of exponentially growing cells of the *WHI5–mCherry MYO1–mCherry* (YMM3056) strain transformed with plasmid pADH1:WHI7–GFP_4_. Disappearance of Myo1 signal (marked with arrowheads) from the neck indicates the end of cytokinesis, referred as to time 0. Approximately 50 cells were analysed with identical results. Dashed lines indicate cell outlines. (D) Exponentially growing cells of the *WHI5–mCherry* (YMM3055), *WHI5–mCherry WHI7–mNeonGreen* (YMM5682) and *WHI5–mNeonGreen* (YMM5680) strains were analysed by confocal fluorescence microscopy. mNeonGreen and mCherry signals are shown. Scale bars: 8 μm (A,D), 4 μm (B), 2 μm (C).

Whi5 subcellular localization is cell cycle regulated. Specifically, Whi5 is located in the nucleus in the G1 phase, from late mitosis (telophase) until Start activation (Costanzo et al., 2004; Taberner et al., 2009). Here, we analysed Whi7 subcellular localization in parallel with that of Whi5. Given the low amount of Whi7 protein, we first used the pADH1:WHI7–GFP_4_ and pADH1:WHI5–GFP_4_ constructs, in which both transcriptional repressors are fused to four copies of the GFP protein and are expressed under the control of the *ADH1* promoter. The analysis of asynchronous cultures confirmed that, like Whi5, the localization of Whi7 changes throughout the cell cycle. Whi7, like Whi5, was located in the nucleus of cells in G1 phase and telophase (Fig. 3A). The assay was also performed in α-factor synchronized cultures. The results indicated that Whi7 was nuclear in G1 cells (0–30 min), disappeared from the nucleus after activation of Start coinciding with budding, and only returned to the nucleus at the end of the cell cycle (150 min) (Fig. 3B). Time-lapse analysis of cells expressing Whi5–mCherry and Whi7–GFP further demonstrated that Whi7 localization is regulated throughout the cell cycle with the same localization kinetics as Whi5 (Fig. 3C; Movie 1). However, it is important to note that in all the experimental approaches, whereas Whi5 was totally nuclear during G1 phase, Whi7 never became completely nuclear, but rather was distributed between the nucleus and the cytosol of the cell. Finally, we investigated Whi7 localization at endogenous levels using the mNeonGreen tag. Notably, the results confirmed the Whi7 cell cycle-regulated pattern described above (Fig. 3D; Movie 2). Whi7 is an unstable protein (Gomar-Alba et al., 2017), so we wondered whether the disappearance of nuclear signal could be due to a selective degradation of nuclear Whi7. However, Whi7 stabilization in a *grr1* mutant strain did not alter the cell cycle-regulated pattern of Whi7 localization (Fig. S1). This result points to the existence of an active nuclear export mechanism controlling Whi7 subcellular localization in a cell cycle-regulated manner.

**Fig. 4.**
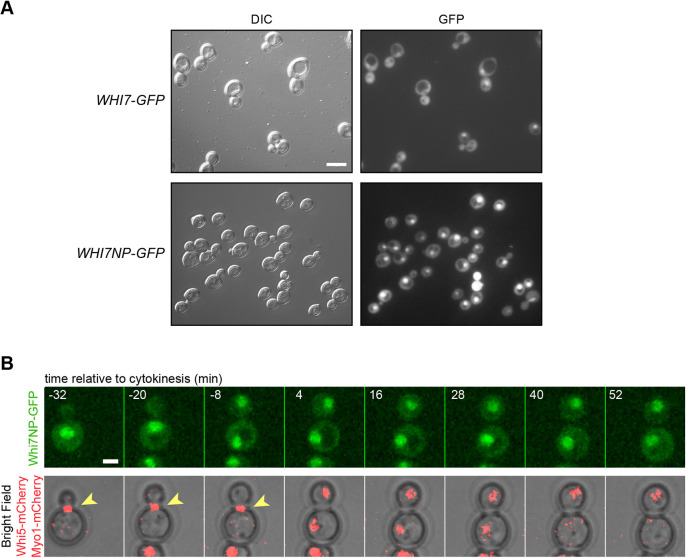
**The non-phosphorylatable Whi7 protein is nuclear in all cell cycle stages.** (A) Exponentially growing cells of the wild-type (W303-1a) strain transformed with either plasmid pADH1:WHI7–GFP_4_ or plasmid pADH1:WHI7NP–GFP_4_, which expresses a Whi7 protein mutated in all consensus CDK phosphorylation sites, were analysed by fluorescence microscopy. GFP signal and DIC images are shown. All cells showed nuclear fluorescence signal (*n*>300 cells from three experiments). (B) Time-lapse analysis by confocal fluorescence microscopy of exponentially growing cells of the *WHI5–mCherry MYO1–mCherry* (YMM3056) strain transformed with plasmid pADH1:WHI7NP–GFP_4_, as described in Fig. 3C. Scale bars: 8 μm (A), 2 μm (B).

Whi7 is a highly phosphorylated protein. In the case of Whi5, the phosphorylation of specific residues by Cdc28 mediates its nuclear export (Costanzo et al., 2004; Taberner et al., 2009; Wagner et al., 2009). For this reason, the localization of Whi7NP, a version of Whi7 protein with all the putative Cdc28 phosphorylation sites mutated to Ala (Yahya et al., 2014), was investigated. The results demonstrated that the non-phosphorylatable Whi7 was nuclear in all cell cycle phases (Fig. 4; Movie 3). This strongly suggests that Cdc28-dependent phosphorylation of Whi7 is necessary for Whi7 nuclear export, as occurs with Whi5.

**Fig. 5.**
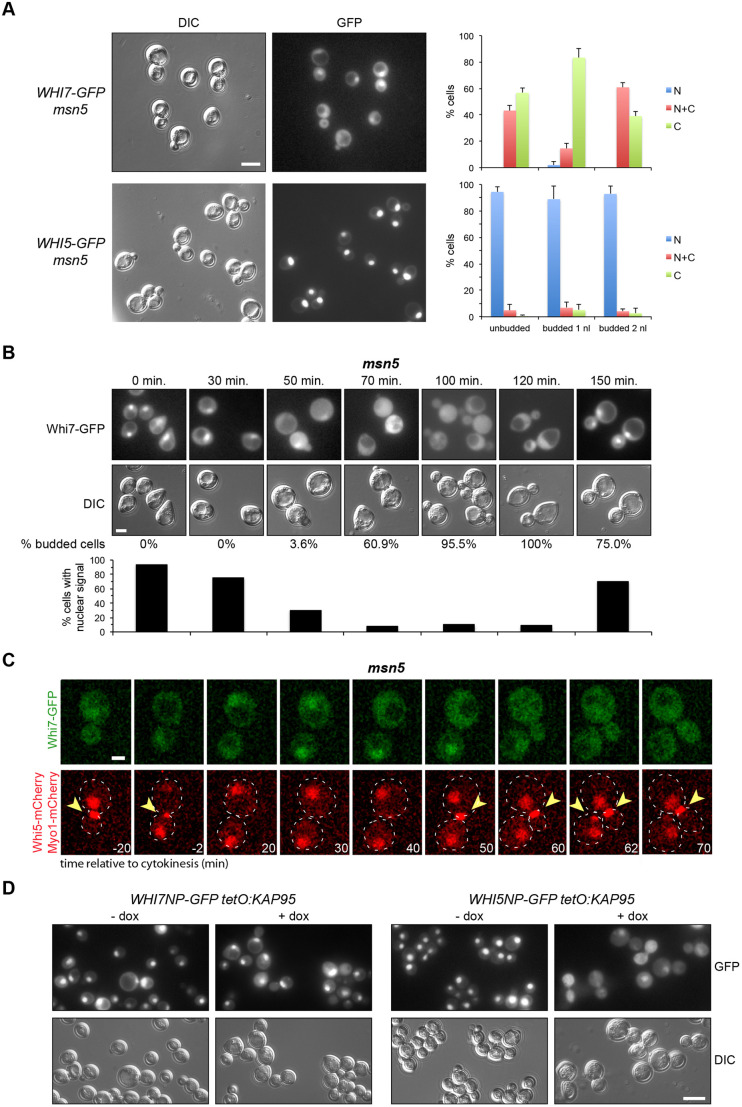
**Msn5 and Kap95 karyopherins control Whi5 but not Whi7 subcellular localization.** (A) Exponentially growing cells of the *msn5* (JCY1018) strain transformed with plasmids pADH1:WHI7–GFP_4_ or pADH1:WHI5–GFP_4_ were analysed by fluorescence microscopy, as described in Fig. 3A. Graphs show mean±s.d. percentage protein localization derived from three (Whi7) or two (Whi5) independent experiments (*n*>300). (B) Whi7 localization was investigated in α-factor synchronized cultures of the *msn5* (JCY1018) strain transformed with plasmid pADH1:WHI7–GFP_4_, as described in Fig. 3B. One of two replicates is shown. (C) Time-lapse analysis by confocal fluorescence microscopy of exponentially growing cells of the *WHI5–mCherry MYO1–mCherry msn5* (YMM5382) strain transformed with plasmid pADH1:WHI7–GFP_4_. as described in Fig. 3C. Arrowheads indicate Myo1–mCherry. Dashed lines indicate cell outlines. (D) Exponentially growing cells of the *tetO_7_:KAP95* strain (JCY970) transformed with plasmid pADH1:WHI7NP–GFP_4_ or pADH1:WHI5NP–GFP_4_ were incubated in the presence of 5 μg/ml doxycycline (+ dox) for 12 h to repress *KAP95* expression. GFP signal and DIC images are shown. Scale bars: 8 μm (A,D), 4 μm (B), 2 μm (C).

Whi5 nuclear export is driven by karyopherin Msn5 through the recognition of a NES (nuclear export signal) in a phosphorylation-dependent manner (Taberner et al., 2009). Given the results described above, we envisaged the possibility that Msn5 could also mediate the nuclear export of Whi7. However, contrary to what happens with Whi5, inactivation of Msn5 did not significantly change Whi7 localization (Fig. 5A–C; Movie 4). We also tested whether the Whi7^70–110^ fragment, covering the equivalent region to the Msn5-dependent NES of Whi5, has NES activity. Unlike what happens with Whi5^51–167^, Whi7^70–110^ was not able to mediate nuclear export when fused to a constitutive nuclear localization protein NLS^SV40^–GFP (Fig. S2). Although it cannot be completely ruled out that Msn5 contributes to Whi7 export (nuclear Whi7 was consistently detected in ∼10% of *msn5* mutant cells outside G1), these results indicate that, in contrast to export of Whi5, Whi7 nuclear export does not require Msn5. Concerning nuclear import, it is known that Whi5 is imported by the classical nuclear import pathway dependent on the Kap60 (Srp1)–Kap95 karyopherin (Taberner et al., 2009). In contrast, inactivation of Kap95 had no effect on the nuclear accumulation of Whi7NP (Fig. 5D). In short, although nuclear localization of Whi7 changed along the cell cycle similarly to that of Whi5, the two proteins use different pathways to control their nuclear localization.

### The PKC pathway controls the cellular levels of Whi7, but not those of Whi5

**Fig. 6.**
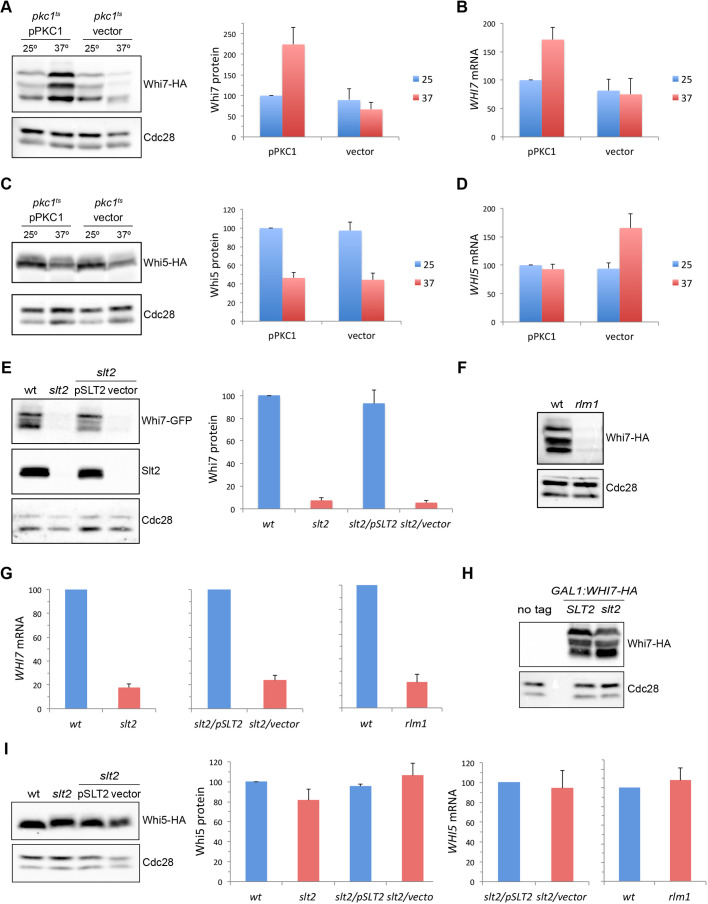
**The PKC pathway regulates expression of *WHI7* but not *WHI5*.** (A,C) Exponentially growing cells of the *WHI7–HA pkc1^ts^* (JCY1730) or *WHI5–HA pkc1^ts^* (JCY2164) strains transformed with plasmid pPKC1 or an empty vector were incubated at 37°C for 3 h. Whi7 (A) or Whi5 (C) protein level was analysed by western blotting. Cdc28 is shown as loading control. Graphs show the mean±s.d. protein levels derived from six (Whi7) or two (Whi5) experiments, normalized to Cdc28. (B,D) The level of *WHI7* (B) or *WHI5* (D) mRNA relative to *ACT1* mRNA was analysed by quantitative RT-PCR in the strains described in A and C. Graphs show the mean±s.d. mRNA level derived from three (Whi7) or four (Whi5) experiments. (E) Whi7 protein level was analysed in exponentially growing cells of *WHI7–GFP* (wt; JCY1746), *WHI7–GFP slt2* (*slt2*; JCY2039) and *WHI7–GFP slt2* (JCY2039) transformed with plasmid pSLT2 or an empty vector. Slt2 and Cdc28 are shown as controls. Graph shows the mean±s.d. Whi7 protein level derived from three experiments, normalized to Cdc28. (F) Whi7 protein level was analysed in exponentially growing cells of *WHI7–HA* (wt; JCY1728) and *WHI7–HA rlm1* (JCY2166) strains. Cdc28 is shown as loading control for protein analysis. (G) *WHI7* mRNA level relative to *ACT1* mRNA level was analysed by quantitative RT-PCR in the same strains described in E and F. Graphs show the mean±s.d. mRNA level derived from four (*slt2*) or three (*rlm1*) experiments. (H) Whi7 protein level was analysed in exponentially growing cells of the wild-type (W303-1a; *SLT2*) and *slt2* (JCY2040) strains transformed with plasmid pGAL1:WHI7–HA, alongside wild-type cells transformed with empty vector (no tag). Cdc28 is shown as loading control for protein analysis. (I) Left: Whi5 protein level was analysed in exponentially growing cells of *WHI5–HA* (wt; JCY1346), *WHI5–HA slt2* (JCY2140) and *WHI5–HA slt2* (JCY2140) transformed with plasmid pSLT2 or an empty vector. Middle: graph shows mean±s.d. protein levels derived from three experiments, normalized to Cdc28. Right: *WHI5* mRNA level relative to *ACT1* mRNA level was analysed by quantitative RT-PCR in the *WHI5–HA slt2* (JCY2140) strain transformed with plasmid pSLT2 or an empty vector and in the wild-type (wt: JCY1728) and *rlm1* mutant (JCY2166) strains. Graphs show the mean±s.d. mRNA level derived from seven (*slt2*) or three (*rlm1*) experiments.

In a preliminary global transcriptomic analysis in a *pkc1* mutant strain, we observed decreased expression of the *WHI7* gene (I.Q. and J.C.I., unpublished), which was consistent with results from other groups connecting *WHI7* gene expression and cell wall stress (Boorsma et al., 2004; Garcia et al., 2004; Lagorce et al., 2003). Because of that, we first investigated Whi7 protein level in a thermosensitive *pkc1* mutant strain (*pkc1^ts^*). As shown in Fig. 6A, there was an increase in Whi7 levels in the wild-type strain at 37°C that was dependent on Pkc1, because it was not observed in the *pkc1* mutant strain at restrictive temperature. The analysis of *WHI7* mRNA demonstrated that this Pkc1-dependent induction probably occurs at the transcriptional level (Fig. 6B).

We extended the analysis to Whi5. Unlike what happens with Whi7, Whi5 levels were reduced at 37°C in wild-type cells (Fig. 6C). The same result was observed after the inactivation of Pkc1. Levels of *WHI5* mRNA were not affected in the wild-type strain at 37°C, whereas they increased approximately twofold in the *pkc1* mutant strain (Fig. 6D). This could be an indirect effect, because *pkc1* mutant cells tend to accumulate in S phase, when the *WHI5* gene has its maximum expression (Pramila et al., 2006). Taken together, these results suggest that Pkc1 activity promotes expression of Whi7 but not expression of its functional paralogue Whi5.

Pkc1 activates the MAP kinase Slt2. It has been previously described that expression of the *WHI7* gene is induced by cell wall stress by Slt2 through the transcription factor Rlm1 (Garcia et al., 2004; Sanz et al., 2012). To address whether the role of Slt2 and Rlm1 in the regulation of *WHI7* expression can be more general, Whi7 levels in *slt2* or *rlm1* mutants were investigated in basal conditions. The Whi7 protein levels were severely reduced in the absence of the MAPK Slt2 or the transcription factor Rlm1 (Fig. 6E,F). In addition, *WHI7* mRNA levels also decreased drastically in the absence of Slt2 or Rlm1 (Fig. 6G). Inactivation of Slt2 had no effect on Whi7 protein level when the *WHI7* gene was ectopically expressed (Fig. 6H). Furthermore, no significant differences in Whi7 protein stability were observed in the *slt2* mutant strain (Fig. S3). These results indicate that, in basal conditions, the MAP kinase Slt2 promotes Whi7 expression through the transcriptional factor Rlm1.

Finally, both Whi5 protein and *WHI5* mRNA levels in the *slt2* and *rlm1* mutant strains were investigated. Contrary to what happens with Whi7, neither the Whi5 protein nor the mRNA levels were altered in the absence of Slt2 or Rlm1 (Fig. 6I). These observations indicate that, unlike what it is observed for Whi7, Whi5 cellular levels are completely independent of the MAP kinase Slt2.

### Cellular regulation and function of Whi7 and Whi5 under cell wall stress

Heat shock stress induces the activation of the PKC pathway, and above, we describe an increase in the cellular levels of Whi7 in this stress condition. We wondered whether this could reflect a more preponderant role for Whi7 compared with that played in non-stress conditions. To test this possibility, we tested the effect of *WHI7* or *WHI5* deletion on the phenotype of a *cln3* mutant strain at high temperature, in which the Start process is compromised. Growth assays revealed that *cln3* mutation was lethal at 38°C and that this lethality was suppressed by either *WHI7* or *WHI5* deletion (Fig. S4). This result suggests, for the first time, that in particular conditions Whi7 could play a major role, comparable at least to that developed by Whi5, in the control of Start.

**Fig. 7.**
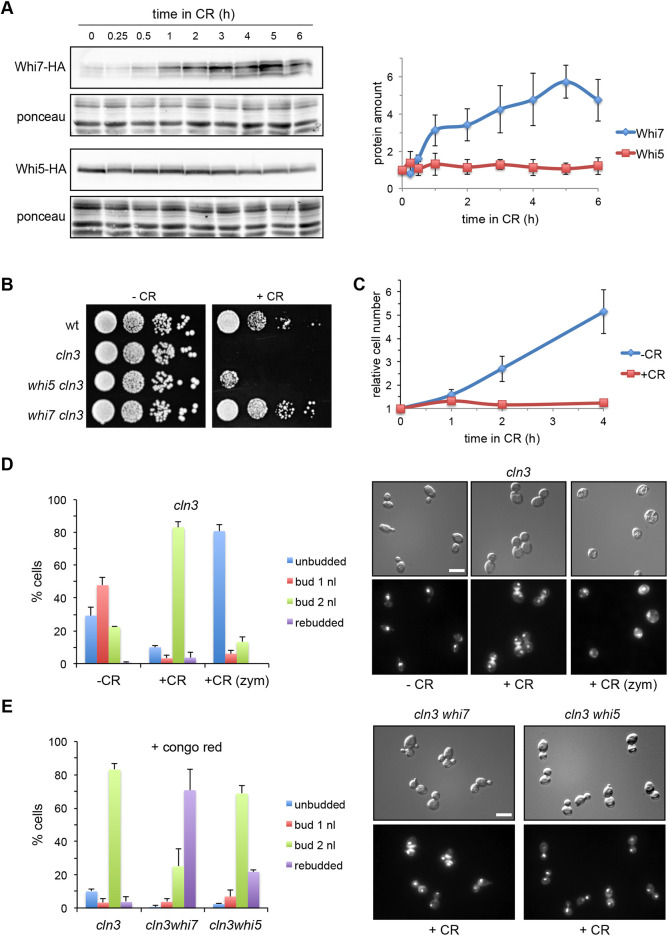
**Whi7 plays a more important role than Whi5 in Start regulation under cell wall stress conditions.** (A) Exponentially growing cells of *WHI7–HA* (JCY2015) or *WHI5–HA* (JCY2036) strains were incubated in the presence of 40 μg/ml Congo Red (CR). Whi7 and Whi5 protein level was analysed by western blotting at the indicated times. The Ponceau staining of the membrane is shown as loading control. Graph shows the mean±s.d. derived from three experiments. (B) Tenfold serial dilutions from cultures of wild-type (wt; W303), *cln3* (MT244), *whi5 cln3* (JCY1875) and *whi7 cln3* (JCY1868) strains were spotted onto YPD plates with or without 40 μg/ml Congo Red and incubated at 25°C for 3 d. Images shown are representative of five experiments. (C) Exponentially growing cultures of the *cln3* (MT244) strain were split and incubated in the absence or presence of 40 μg/ml Congo Red. Cell number was determined at the indicated times. Graph shows the mean±s.d. derived from three experiments. (D) Cell cycle distribution of *cln3* (MT244) cells at 4 h after the addition of Congo Red (+CR). Untreated cells are shown as a control (−CR). Fixed cells were also treated with zymolyase (zym) to digest the cell wall. Cells were scored as unbudded, budded with one nucleus (1nl), budded with two nuclei (2nl) or re-budded. Graph shows the mean±s.d. percentage of cells in each category derived from three experiments. Pictures show DIC images (top) and DAPI staining of DNA (bottom). In the +CR fluorescence images, note the presence of Congo Red fluorescence in the neck associated with a cell separation defect. (E) Cell cycle distribution of *cln3* (MT244), *whi5 cln3* (JCY1875) and *whi7 cln3* (JCY1868) cells at 4 h after the addition of Congo Red. Cells were scored as described in D. Graph shows the mean±s.d. percentage of cells in each category derived from three experiments. Pictures show DIC images (top) and DAPI staining of DNA (bottom). Scale bars: 8 μm.

To further compare Whi7 and Whi5 cellular function under cell wall stress conditions, the effect of Congo Red, a dye that binds to chitin, generating cell wall stress and thus activating the PKC pathway, was studied. Congo Red induces expression of the *WHI7* gene (Garcia et al., 2004). Given this, we tested the effect of Congo Red on the protein levels of Whi7 and Whi5, as well as the effect of this compound on the stability of the Whi7 protein. In line with previous studies of gene expression, Whi7 protein levels increased more than fourfold in the presence of Congo Red (Fig. 7A). The same result was observed with another cell wall stressor, Calcofluor White (Fig. S5A). Translational shut-off assays revealed that Whi7 protein stability was not altered in response to Congo Red (Fig. S6), which supports Whi7 induction being a transcriptional response. No change in Whi7 subcellular localization after cell wall stress was observed (Fig. S5B). Interestingly, in contrast to what occurs with Whi7, Whi5 protein levels were not affected by cell wall stress.

Next, the effect of Congo Red on cell growth in wild-type, *whi7* mutant and *whi5* mutant strains was studied. As observed in Fig. S7A, growth of the wild-type strain was impaired in the presence of Congo Red. Microscopic analysis of the cells revealed a significant increase in the percentage of dumbbell cells (cells with large bud and segregated nuclei) in Congo Red-containing medium. Remarkably, digestion with zymolyase led to the disappearance of dumbbell cells and an increase in the percentage of unbudded cells (Fig. S7B). This indicated that dumbbell cells completed mitosis but had a defect in cell separation caused by the Congo Red. The accumulation of cells in the G1 phase suggests a partial blockage of Start in response to cell wall stress. Interestingly, both the *whi7* and *whi5* mutations alleviated, to a similar extent, the growth defect of wild-type cells in Congo Red medium (Fig. S7A). However, microscopic analysis revealed that Whi7 inactivation suppressed the accumulation of cells in G1 to a greater extent than Whi5 inactivation (Fig. S7B). This points to Whi7 transcriptional repressor as an important mediator of the partial repression of Start caused by Congo Red-induced cell wall stress.

Whi7 represses transcription of the G1/S genes in an SBF- and Swi4-dependent manner. For this reason, we investigated the requirement of Swi4 and Swi6 for Whi7 function in response to CR. It is known that *swi4* and *swi6* mutants are hypersensitive to Congo Red (Garcia et al., 2004). Growth assays showed that deletion of *WHI7* or *WHI5* did not suppress the lethality of the *swi4* and *swi6* mutations in the presence of Congo Red (Fig. S8). This result strongly suggests that Whi7 function in response to Congo Red is dependent on Swi4 and Swi6 activity.

Finally, we studied the role of Whi7 and Whi5 during cell wall stress in a *cln3* mutant background, in which the Start process is compromised. Note that the *cln3* mutation was lethal in the presence of Congo Red (Fig. 7B). Analysis of exponentially growing cultures revealed a first-cycle arrest of *cln3* cells after the addition of Congo Red to the medium (Fig. 7C). Practically all of the *cln3* mutant cells were dumbbell cells (Fig. 7D). Zymolyase treatment indicated that these cells were in fact in G1 phase. All these data reflect that the lethality of *cln3* mutation in the presence of Congo Red is due to a robust arrest in G1, before Start is executed. Importantly, the lethality of the *cln3* mutation in the presence of Congo Red was completely abolished by the deletion of *WHI7*. In contrast, deletion of *WHI5* had a very slight effect on growth (Fig. 7B). The same result was observed in the presence of Calcofluor White (Fig. S5C). In addition, microscopic analysis of *cln3 whi7* cells in the presence of Congo Red confirmed that the absence of Whi7 overrides the G1 arrest of the *cln3* mutant, as revealed by the increase in the percentage of re-budded cells. However, and consistent with what was observed in the growth assay, loss of Whi5 only allowed a small percentage of cells to re-bud (Fig. 7E). In conclusion, all these observations demonstrate that the *cln3* mutant suffers a cell cycle arrest in G1 phase, mediated by the Start transcriptional repressor Whi7 and not by Whi5. Thus, whereas Whi5 is the main Start inhibitor during normal growth, Whi7 takes over this role specifically during cell wall stress.

## DISCUSSION

In a previous work, our group characterized that the Whi7 protein, which is related at sequence level to the transcriptional repressor Whi5 (the functional paralogue of the Rb proteins in mammalian cells) and functions as a transcriptional repressor of the Start transcriptional programme, demonstrating the existence of functional redundancy between Whi7 and Whi5 (Gomar-Alba et al., 2017). However, this does not necessarily mean that both proteins perform exactly the same function or are regulated in the same way. In fact, Whi5 is more critical than Whi7 for proper execution of the Start transition in normal conditions, as deduced from the fact that Whi5, but not Whi7, inactivation leads to a decrease in cell size distribution in exponentially growing cultures, and *WHI5*, but not *WHI7*, overexpression results in a severe increase in cell size. In this work, the differences between Whi7 and Whi5 have been analysed in detail, with the aim of establishing the determinants of functional specificity.

Two possible, non-mutually exclusive scenarios can be considered in order to explain functional differences between redundant proteins that collaborate in a cellular process: a quantitative model, which points to differences in protein level as the origin of functional specificity, and a qualitative model, which proposes the existence of differential intrinsic characteristics. Comparative analysis of cellular levels reveals that Whi5 is in excess over Whi7 (Gomar-Alba et al., 2017). This could explain some functional differences, such as the fact that Whi5 is the repressor that plays the most important role in the control of Start in normal conditions. We have previously reported that *WHI7* overexpression rescues the cell size defect of a *whi5* mutant (Gomar-Alba et al., 2017), which could support this idea. However, it is necessary to consider that this overexpression was accomplished using the very strong *GAL1* promoter. On the other hand, *WHI7* overexpression does not lead to its increased binding to the *CLN2* promoter (Gomar-Alba et al., 2017). These observations could reflect that the functional specificity of these proteins is not only due to quantitative differences but also to the existence of some differential intrinsic characteristics.

A detailed analysis of Whi7 and Whi5 promoter association demonstrated that Whi7 shares with Whi5 the association with the promoters of all analysed genes: Start cyclin genes (*CLN2* and *CLN1*), cell wall genes (*FKS1*, *MNN1* and *GAS1*) and DNA metabolism genes (RNR1). Importantly, ChIP experiments in which both Whi7 and Whi5 were immunoprecipitated from the same cells showed a distinct binding pattern to these promoters. Whereas Whi5 had the strongest preference for G1 cyclin genes, Whi7 preference was greater for cell wall biosynthesis genes. The fact that Whi7 and Whi5 were distributed differently among the promoters is an important result that reveals the existence of differential determinants in their promoter association.

The analysis of Whi7 and Whi5 association with promoters in several mutant strains reflects a fundamental difference in their function. Consistently with previous results, Whi5 is associated with promoters through the intact SBF heterodimer transcriptional factor, because no association was detected in either a *swi6* or a *swi4* mutant. However, in the case of Whi7, binding to promoters was still observed in the absence of Swi6. Different observations support that this association is mediated by Swi4 alone. It has been described that monomeric Swi4 is the unique Start transcription factor active in *swi6* mutant cells (Adames et al., 2015; Nasmyth and Dirick, 1991; Wijnen et al., 2002). In fact, we detected Swi4 associated with the same promoters as Whi7 in *swi6* mutant cells. Remarkably, we observed stronger Whi7 binding in *swi6* cells in the case of the *FKS1* gene, a gene for which the binding of Swi4 was barely affected by the inactivation of Swi6. In addition, Whi7, but not Whi5, was capable of physically interacting *in vivo* with Swi4 in the absence of Swi6. These results reveal a key difference between Whi7 and Whi5 as transcriptional repressors of Start, because, unlike Whi5, Whi7 represses the expression of G1/S genes, not only through SBF, but also through monomeric Swi4 (Fig. 2F). Considering that monomeric Swi4 can function as a transcriptional activator that evades Whi5-mediated regulation, this specific characteristic of Whi7 may be relevant for cellular physiology.

We noted that some residual binding of Whi7 to promoters was still observed in the *swi4* mutant. Although Whi7 binding to promoters in the wild-type strain did not depend on Mbp1, the fact that, in the absence of Swi4, inactivation of Mbp1 reduced promoter binding strongly suggests that Whi7 could also associate with promoters through the MBF transcriptional factor. Consistent with this, Mbp1 was able to bind to the same promoters as Whi7 in the absence of Swi4. Remarkably again, the strongest binding of Whi7 in the absence of Swi4 was observed for the case of the *FKS1* gene, which among the genes we examined showed the greatest association with Mbp1 in the absence of Swi4. However, we could not confirm a physical interaction between Whi7 and Mbp1 in co-immunoprecipitation assays. From our results on the effect of *swi4* and *mbp1* mutations on Whi7 promoter association, it can be expected that the interaction between Whi7 and MBF might be weak and secondary, which could explain the failure to detect it. A known repressor of MBF is Nrm1, the third member of the family of GTB-motif G1/S transcriptional repressors (Travesa et al., 2013). Unlike Whi7 and Whi5, Nrm1 mediates repression of MBF after Start execution, once it has been transcribed by MBF itself (de Bruin et al., 2006). The possibility that Whi7 can repress MBF before Start brings into light an additional mechanism to control this transcriptional programme (Fig. 2F). As commented above for the role of Whi7 on monomeric Swi4, this new mechanism could have biological relevance under certain conditions, for example when it is necessary to block the MBF factor before Start.

Spatial regulation is a common mechanism in the control of transcription factors. It is known that Whi5 shuttles between the nucleus and cytoplasm throughout the cell cycle, being located in the nucleus from mitotic exit until the G1-S transition and accumulating in the cytoplasm during the rest of the cell cycle phases (Costanzo et al., 2004; Taberner et al., 2009). In this study, we have described that the subcellular localization of Whi7 is also cell cycle-regulated and follows the same dynamics as Whi5, that is, Whi7 is nuclear only in the G1 phase until Start. However, there is an important difference; Whi5 totally accumulates inside the nucleus in G1, whereas Whi7 is distributed between the nucleus and the cytosol. Consistent with this cytosolic localization, Whi7 plays a function in G1 bound to the ER membrane (Yahya et al., 2014). The determinants responsible for Whi5 spatial regulation are known. Whi5 nuclear export is dependent on the karyopherin Msn5 and the phosphorylation by Cdc28 of specific residues in a nuclear export signal, and nuclear import is mediated by the classical nuclear import pathway (Taberner et al., 2009). We have identified that Whi7 nuclear export is also controlled by Cdc28-dependent phosphorylation. However, in contrast to Whi5, Whi7 nuclear export did not require the Msn5 karyopherin and Whi7 nuclear import did not require the classical nuclear import pathway (Kap60–Kap95 karyopherin). Thus, Whi7 and Whi5 differ in the way their subcellular location is controlled. This difference could provide additional ways to differentially regulate both repressors under specific conditions.

Previous work has found a relationship between the CWI pathway and expression of the *WHI7* gene. Global studies have shown an induction of the *WHI7* gene in different conditions that affect cell wall integrity (Boorsma et al., 2004; Garcia et al., 2004; Lagorce et al., 2003). Moreover, the expression of *WHI7* is induced by the presence of Congo Red in a Slt2-dependent manner (Sanz et al., 2012). In this work, we gained further insight into the characterization of *WHI7* gene regulation by the PKC pathway. In particular, we found that Slt2, through the transcriptional factor Rlm1, is responsible for the expression of the *WHI7* gene under basal conditions. It should be remarked that Whi7 expression is practically abolished in the *slt2* and *rlm1* mutants. This points to a very close functional connection between the MAPK Slt2 and the Start transcriptional repressor Whi7, with Whi7 function totally dependent on Slt2. Conversely, Slt2 is activated by distinct stimuli, and these results point to Whi7 as a mediator of the cellular response to these stimuli. Slt2, through Rlm1, regulates the expression of cell wall genes (Sanz et al., 2017). On the other hand, a connection between Slt2 and the components of SBF, Swi4 and Swi6, has been previously described (Baetz et al., 2001; Kim et al., 2008; Truman et al., 2009), and SBF is involved in the expression of cell wall genes (Igual et al., 1996). All these considerations suggest that the function of Whi7 as a repressor of Start could be more relevant under certain conditions, such as cell wall stress, in which there is a higher activity of the PKC pathway. Our results provide support for this hypothesis: *WHI7* deletion suppressed the sensitivity and G1 arrest of the *cln3* mutant in response to elevated temperatures and to compounds that stress the cell wall. The fact that Whi7 inactivation did not suppress the hypersensitivity of *swi4* and *swi6* mutants to Congo Red indicates that Whi7 acts under cell wall stress conditions through SBF. Interestingly, in contrast to what was observed with Whi7, the PKC pathway did not affect the cellular levels of Whi5, establishing a new key difference between both transcriptional repressors. Consistent with this, *WHI7* deletion had a greater effect than *WHI5* deletion on cell growth at high temperature or in the presence of Congo Red or Calcofluor White. Overall, our results indicate that in response to cell wall stress, it is Whi7 and not Whi5 transcriptional repressor that plays a major role as a brake on cell cycle entry at Start. Whi7 induction in the presence of cell wall stress could contribute to this, but the increase in Whi7 cellular content seems quite modest in relation to Whi5 protein level to support its major role. Therefore, how this functional switch could operate is still an open question.

**Fig. 8.**
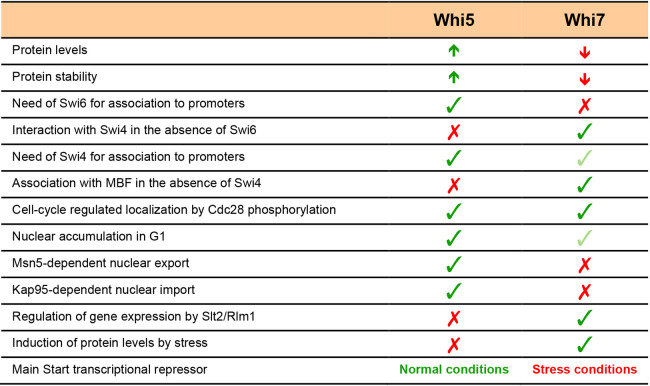
**Comparison between Whi7 and Whi5.** Differences between the budding yeast Start repressors Whi5 and Whi7 regarding protein level, determinants in binding to promoters, control of subcellular localization, regulation of gene expression and response to stress condition are listed.

Functional redundancy between proteins is a common trait in cell cycle regulation. One good example is the existence of several transcriptional repressors at the heart of Start control: Rb, p107 and p130 in mammalian cells, and Whi5 and Whi7 in *S. cerevisiae* cells. However, despite the functional redundancy that exists between the Start transcriptional repressors, we have described in this work that Whi7 and Whi5 greatly differ both in their regulation and in their functionality (Fig. 8). The differential characteristics and regulation of Whi7 compared to Whi5 could contribute to a more robust control of Start under specific circumstances. On one hand, the intrinsic different requirements for Whi7 binding to promoters confers new repression capability of Start genes beyond Whi5-mediated repression, in particular as regards the regulation of the Swi4 transcription factor, which could activate G1/S genes on its own, and MBF before Start. On the other hand, the differential regulation of Whi7 and Whi5 provides alternative ways to affect Start repression depending on internal or external cues. This allows yeast cells to rely on the interplay between distinct Start transcriptional repressors to adapt to different specific cellular conditions: Whi5 stands out as the main transcriptional repressor under normal conditions, whereas the function of Whi7 becomes more relevant in stress conditions, such as cell wall stress. The existence of multiple repressor proteins for the Start transcriptional programme must certainly represent an evolutionary advantage for the cell.

## MATERIALS AND METHODS

### Strains, plasmids and growth conditions

Yeast strains used in this work are shown in Table S1. Centromeric plasmid pWHI7-HA expressing a 3×HA-tagged Whi7 protein under the control of the endogenous promoter is a gift from Dr M. Aldea, Molecular Biology Institute of Barcelona (IBMB), Spain (Yahya et al., 2014). pADH1:WHI7–GFP_4_, pADH1:WHI7NP–GFP_4_, pADH1:WHI5–GFP_4_ and pADH1:WHI5NP–GFP_4_ contain the *ADH1* promoter, the coding region of *WHI7* and *WHI5* and their respective mutations in canonical CDK phosphorylation sites fused to four copies of GFP and the *ADH1* terminator cloned in the YCplac33 vector (Gietz and Sugino, 1988). The *WHI7NP* and *WHI5NP* coding region were obtained from plasmid pWHI7NP–HA (Yahya et al., 2014) and pGAL1:WHI5^12Ala^–13myc (Wagner et al., 2009). pADH1:NLS–WHI7^70–110^–GFP_4_ and pADH1:NLS–WHI5^51–167^–GFP_4_ contain the *ADH1* promoter, a fragment coding for the nuclear localization sequence from SV40 and either a *WHI7* gene fragment coding for amino acids 70–110 or a *WHI5* gene fragment coding for amino acids 51–167 fused to four copies of GFP and the *ADH1* terminator in YCplac33 vector. pPKC1 and pSLT2 consist of the *PKC1* or *SLT2* gene cloned in YCplac33.

Cells were maintained in exponential growth conditions (below 10^7^ cells ml^−1^) at 25°C in standard YPD medium (1% yeast extract, 2% peptone and 2% glucose) or synthetic complete medium lacking the appropriate compound for selection (Formedium Ltd) supplemented with 2% glucose (SD) or 2% galactose (SGal). Where indicated, cells were incubated in the presence of 40 μg ml^−1^ Congo Red, 10 μg ml^−1^ Calcofluor White or transferred to 37°C for 3 h. For cell cycle synchronization, *MATa* cells were arrested in G1 with 5 μg/ml of α-factor for 3 h and then released into fresh growth medium.

### Fluorescence microscopy

GFP, mCherry and mNeonGreen tagged proteins were analysed in living cells grown on synthetic complete medium. Conventional fluorescence microscopy was carried out with an Axioskop 2 microscope (Zeiss). The images were captured with an AxioCam MRm camera (Zeiss) and AxioVision v4.7 software (Zeiss). Where indicated, localization of proteins was monitored by visual inspection.

Confocal fluorescence microscopy was carried out on a confocal spinning-disk microsope (Nikon Ti) equipped with an HCX plan APO 100× objective and a Photometrics Prime 95B camera. Four to eight *z*-sections, 0.35 μm apart, were collected every 90 or 120 s at 30°C. Images in the figures and movies are 2D maximum projections of the *z*-stacks. Images were processed using ImageJ (http://rsb.info.nih.gov/ij/). For time-lapse microscopy, exponentially growing cells (below 10^7^ cells ml^−1^) were grown in synthetic complete medium and plated on concanavalin A-coated (Sigma-Aldrich) Lab-Tek chambers (Thermo Fisher Scientific). Temperature during the images acquisition was controlled with a Tokai Hit Stage Top Incubator.

For DNA staining, cells were fixed for 5 min by addition of 70% ethanol and resuspended in 1 μg/ml DAPI (4′,6-diamidino-2-phenylindole). Where indicated, the cell wall was digested in fixed cells using 0.5 mg/ml zymolyase 20T (SEIKAGAKU CORPORATION) in phosphate spheroplasting buffer (1.2 M sorbitol, 0.1% β-mercaptoethanol and 50 mM KH_2_PO_4_, pH 7.0) at 30°C for 30 min.

### Miscellaneous

Chromatin immunoprecipitation analysis (ChIP), gene expression analysis by RT-PCR, protein co-immunoprecipitation assays and western blot analysis were performed as previously described (Gomar-Alba et al., 2017). In ChIP assays, Dynabeads Protein G magnetic beads were incubated with HA-probe (F-7) antibody (Santa Cruz Biotechnology; SC-7392) or monoclonal anti-GFP (Roche Diagnostics; 11814460001). The primary antibodies used in the western blot analysis were monoclonal anti-HA peroxidase 3F10 antibody (Roche Diagnostics; 12013819001) diluted 1:5000, monoclonal anti-GFP (Roche Diagnostics; 11814460001) diluted 1:5000 and monoclonal anti Cdc2 p34 (PSTAIRE; Santa Cruz Biotechnology; SC-53) diluted 1:2000. Blots were developed using anti-mouse IgG and anti-rabbit IgG horseradish peroxidase-conjugated secondary antibodies (1:20,000; Thermo Fisher Scientific; 170-6516 and 31460, respectively) and Supersignal West Femto Maximum Sensitivity substrate (Thermo Fisher Scientific). Bands were quantified using an ImageQuant LAS 4000mini Biomolecular Imager (GE Healthcare).

## Supplementary Material

Supplementary information

Reviewer comments
