# Supplementary material for: The budding yeast Start repressor Whi7 differs in regulation from Whi5, emerging as a major cell cycle brake in response to stress
Source: J Cell Sci. 2020 Dec 21;133(24):jcs251413. doi: 10.1242/jcs.251413 (PMC7774886; doi:10.1242/jcs.251413)
Supplement: Supplementary information [file joces-133-251413-s1.pdf]

Supplemental Table S1. Yeast strains

W303-1a	*MATa ade2-1 trp1-1 leu2-3,112 his3-11,15 ura3-52 can1*
S288C	*Mata ura3-52 his3Δ200 leu2 lys2-801 ade2-101 trp1Δ63*
MT244	*MATa ade2-1 trp1-1 leu2-3,112 his3-11,15 ura3-52 can1 cln3::URA3*
cdc15^{ts}	*MATa ade2-1 trp1-1 leu2-3,112 his3-11,15 ura3-52 can1 cdc15-2*
JC6-3A	*MATa rsf8/pkc1-8 ade2 ura3 his3 leu2 trp1 can1 met*
JCY0167	*MATa ade2-1 trp1-1 leu2-3,112 his3-11,15 ura3-52 can1 swi4::LEU2* (*swi4::LEU2* in W303-1a)
JCY0221	*MATa ade2-1 trp1-1 leu2-3,112 his3-11,15 ura3-52 can1 swi6::TRP1* (*swi6::TRP1* in W303-1a)
JCY0325	*MATa ade2-1 trp1-1 leu2-3,112 his3-11,15 ura3-52 can1 swi6::kanMX6* (*swi6::kanMX6* in W303-1a)
JCY0740	*MATa ade2-1 trp1-1 leu2-3,112 his3-11,15 ura3-52 can1 swi4::LEU2 WHI5-3HA-TRP1* (*WHI5-3HA-TRP1* en JCY0167)
JCY0970	*MATa ade2-1 trp1-1 leu2-3,112 his3-11,15 ura3-52 can1 tTR´::LEU2 tetO$_7$:KAP95-kanMX6* (*tTR´::LEU2 tetO$_7$:KAP95-kanMX6* in W303-1a)
JCY1018	*MATa ade2-1 trp1-1 leu2-3,112 his3-11,15 ura3-52 can1 msn5::HIS3* (*msn5::HIS3* in W303-1a)
JCY1346	*MATa ade2-1 trp1-1 leu2-3,112 his3-11,15 ura3-52 can1 WHI5-HA-TRP1* (*WHI5-HA-TRP1* in W303-1a)
JCY1539	*MATa ade2-1 trp1-1 leu2-3,112 his3-11,15 ura3-52 can1 grr1::LEU2* (*grr1::LEU2* in W303-1a)
JCY1728	*MATa ade2-1 trp1-1 leu2-3,112 his3-11,15 ura3-52 can1 WHI7-HA-TRP1* (*WHI7-HA-TRP1* in W303-1a)
JCY1730	*MATa rsf8/pkc1-8 ade2 ura3 his3 leu2 trp1 can1 met WHI7-HA-TRP1* (*WHI7-HA-TRP1* in JC6-3A)
JCY1746	*MATa ade2-1 trp1-1 leu2-3,112 his3-11,15 ura3-52 can1 WHI7-GFP-kanMX6* (*WHI7-GFP-kanMX6* in W303-1a)
JCY1819	*MATa ade2-1 trp1-1 leu2-3,112 his3-11,15 ura3-52 can1 whi7::kanMX6* (*whi7::kanMX6* in W303-1a)
JCY1868	*MATa ade2-1 trp1-1 leu2-3,112 his3-11,15 ura3-52 can1 cln3::URA3 whi7::kanMX6* (*whi7::kanMX6* in MT244)
JCY1872	*MATa ade2-1 trp1-1 leu2-3,112 his3-11,15 ura3-52 can1 swi4::LEU2 whi7::kanMX6* (*whi7::kanMX6* in JCY0167)
JCY1874	*MATa whi5::LEU2 ade2-1 trp1-1 leu2-3,112 his3-11,15 ura3-52 can1* (*whi5::LEU2* in W303-1a)
JCY1875	*MATa ade2-1 trp1-1 leu2-3,112 his3-11,15 ura3-52 can1 cln3::URA3 whi5::LEU2* (*whi5::LEU2* in MT244)
JCY1879	*MATa ade2-1 trp1-1 leu2-3,112 his3-11,15 ura3-52 can1 cdc15-2 SWI4-13myc-HIS3* (*SWI4-13myc-HIS3* in *cdc15^{ts}*)
JCY1880	*MATa ade2-1 trp1-1 leu2-3,112 his3-11,15 ura3-52 can1 swi6::TRP1 whi7::kanMX6* (*whi7::kanMX6* in JCY0221)
JCY1884	*MATa cdc15-2 ade2-1 trp1-1 leu2-3,112 his3-11,15 ura3-52 can1 SWI4-13myc-HIS3 swi6::kanMX6*

	(*swi6::kanMX6* in JCY1879)
JCY1934	*MATa ade2-1 trp1-1 leu2-3,112 his3-11,15 ura3-52 can1 swi6::TRP1 WHI5-3HA-kanMX6*<br>(*WHI5-3HA-kanMX6* in JCY0221)
JCY1952	*MATa ade2-1 trp1-1 leu2-3,112 his3-11,15 ura3-52 can1 WHI7-GFP-TRP1*<br>(*WHI7-GFP-TRP1* in W303-1a)
JCY1956	*MATa ade2-1 trp1-1 leu2-3,112 his3-11,15 ura3-52 can1 WHI7-GFP-TRP1 SWI4-6HA-HIS3*<br>(*SWI4-6HA-HIS3* in JCY1952)
JCY1958	*MATa ade2-1 trp1-1 leu2-3,112 his3-11,15 ura3-52 can1 WHI7-GFP-TRP1 SWI4-6HA-HIS3 swi6::kanMX6*<br>(*swi6::kanMX6* in JCY1956)
JCY2015	*MATa ade2-1 trp1-1 leu2-3,112 his3-11,15 ura3-52 can1 WHI7-6HA-HIS3*<br>(*WHI7-6HA-HIS3* in W303-1a)
JCY2036	*MATa ade2-1 trp1-1 leu2-3,112 his3-11,15 ura3-52 can1 WHI5-6HA-HIS3*<br>(*WHI5-6HA-HIS3* in W303-1a)
JCY2039	*MATa ade2-1 trp1-1 leu2-3,112 his3-11,15 ura3-52 can1 WHI7-GFP-kanMX6 slt2::HIS3*<br>(*slt2::HIS3* in JCY1746)
JCY2040	*MATa ade2-1 trp1-1 leu2-3,112 his3-11,15 ura3-52 can1 slt2::HIS3*<br>(*slt2::HIS3* in W303-1a)
JCY2084	*MATa ade2-1 trp1-1 leu2-3,112 his3-11,15 ura3-52 can1 MBP1-6HA-HIS3*<br>(*MBP1-6HA-HIS3* in W303-1a)
JCY2095	*MATa ade2-1 trp1-1 leu2-3,112 his3-11,15 ura3-52 can1 swi6::TRP1 whi5::kanMX6*<br>(*whi5::kanMX6* in JCY0221)
JCY2100	*MATa ade2-1 trp1-1 leu2-3,112 his3-11,15 ura3-52 can1 MBP1-6HA-HIS3 swi4::LEU2*<br>(*swi4::LEU2* in JCY2084)
JCY2116	*MATa ade2-1 trp1-1 leu2-3,112 his3-11,15 ura3-52 can1 WHI5-GFP-kanMX6*<br>(*WHI5-GFP-kanMX6* in W303-1a)
JCY2134	*MATa cdc15-2 ade2-1 trp1-1 leu2-3,112 his3-11,15 ura3-52 can1 SWI4-13myc-HIS3 WHI5-3HA-TRP1*<br>(*WHI5-3HA-TRP1* in JCY1879)
JCY2135	*MATa cdc15-2 ade2-1 trp1-1 leu2-3,112 his3-11,15 ura3-52 can1 SWI4-13myc-HIS3 WHI5-3HA-TRP1 swi6::kanMX6*<br>(*swi6::kanMX6* in JCY2134)
JCY2237	*MATa ade2-1 trp1-1 leu2-3,112 his3-11,15 ura3-52 can1 swi4::LEU2 whi5::kanMX6*<br>(*whi5::kanMX6* in JCY0167)
JCY2140	*MATa ade2-1 trp1-1 leu2-3,112 his3-11,15 ura3-52 can1 WHI5-HA-TRP1 slt2::HIS3*<br>(*slt2::HIS3* in JCY1346)
JCY2164	*MATa rsf8/pkc1-8 ade2 ura3 his3 leu2 trp1 can1 met WHI5-6HA-HIS3*<br>(*WHI5-6HA-HIS3* in JC6-3A)
JCY2166	*MATa ade2-1 trp1-1 leu2-3,112 his3-11,15 ura3-52 can1 WHI7-HA-TRP1 rlm1::LEU2*<br>(*rlm1::LEU2* in JCY1728)
JCY2194	*MATa ade2-1 trp1-1 leu2-3,112 his3-11,15 ura3-52 can1 mbp1::HIS3*<br>(*mbp1::HIS3* in W303-1a)
JCY2268	*MATa ade2-1 trp1-1 leu2-3,112 his3-11,15 ura3-52 can1 GAL1:SWI4::kanMX6*<br>(*GAL1:SWI4::kanMX6* in W303-1a)
JCY2304	*MATa ade2-1 trp1-1 leu2-3,112 his3-11,15 ura3-52 can1 GAL1:SWI4::kanMX6 mbp1::HIS3*<br>(*mbp1::HIS3* in JCY2268)

YMM3055	*MATa ura3-52 his3Δ200 leu2 lys2-801 ade2-101 trp1Δ63 WHI5-mCherry-NAT* (*WHI5-mCherry-NAT* in S288C)
YMM3056	*MATa ura3-52 his3Δ200 leu2 lys2-801 ade2-101 trp1Δ63 WHI5-mCherry-NAT MYO1-mCherry-HYG* (*MYO1-mCherry-HYG* in YMM3055)
YMM5382	*MATa ura3-52 his3Δ200 leu2 lys2-801 ade2-101 trp1Δ63 WHI5-mCherry-NAT MYO1-mCherry-HYG msn5::kanMX6* (*msn5::kanMX6* in YMM3056)
YMM5680	*MATa ura3-52 his3Δ200 leu2 lys2-801 ade2-101 trp1Δ63 WHI5-ymNeonGreen-URA3* (*WHI5-ymNeonGreen-URA3* in S288C)
YMM5682	*MATa ura3-52 his3Δ200 leu2 lys2-801 ade2-101 trp1Δ63 WHI5-mCherry-NAT WHI7-ymNeonGreen-URA3* (*WHI7-ymNeonGreen-URA3* in YMM3055)

Journal of Cell Science • Supplementary information

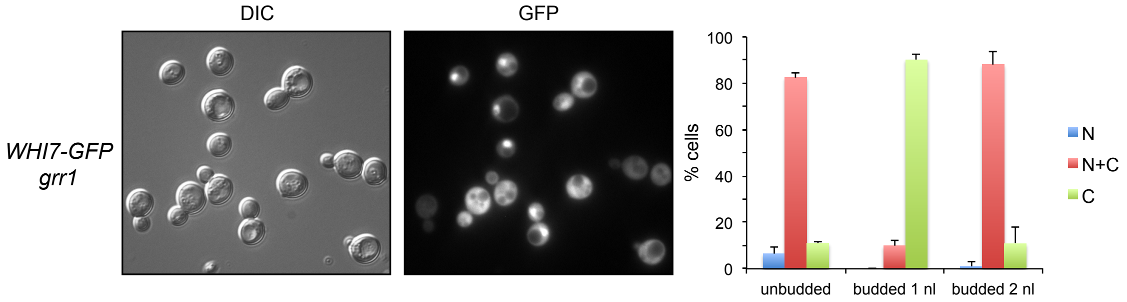

Figure S1. Subcellular localization of Whi7 is not altered in the *grr1* mutant strain. Exponentially growing cells of the *grr1* (JCY1539) strain transformed with plasmid pADH1:WHI7-GFP$_4$ were analysed by fluorescence microscopy. GFP signal and DIC images are shown. Cells were scored as unbudded, budded with one nucleus (1nl) or budded with two nuclei (2nl), and as cells with fluorescence signal only in the nuclei (N), in the nuclei and the cytoplasm (N+C) or only in the cytoplasm (C). Graphs represent protein localization derived from three independent experiments (n>300).

Journal of Cell Science • Supplementary information

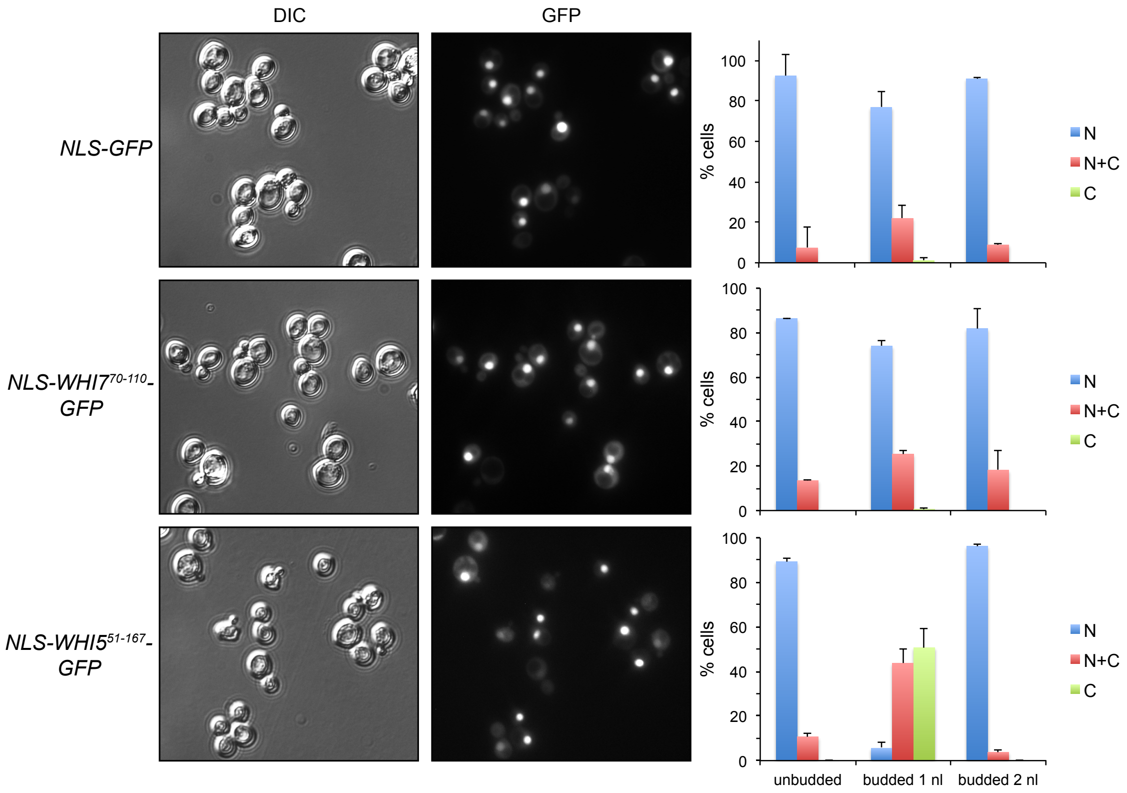

Figure S2. Whi7 region equivalent to Whi5 NES has not nuclear export function. Exponentially growing cells of the wild type (W303-1a) strain transformed with plasmids pADH1:NLS-GFP$_4$, pADH1:NLS-WHI7$^{70\text{-}110}$-GFP$_4$ or pADH1:NLS-WHI5$^{51\text{-}167}$-GFP$_4$ were analysed by fluorescence microscopy. GFP signal and DIC images are shown. Cells were scored as unbudded, budded with one nucleus (1nl) or budded with two nuclei (2nl), and as cells with fluorescence signal only in the nuclei (N), in the nuclei and the cytoplasm (N+C) or only in the cytoplasm (C). Graphs represent protein localization derived from three independent experiments (n>300).

Journal of Cell Science • Supplementary information

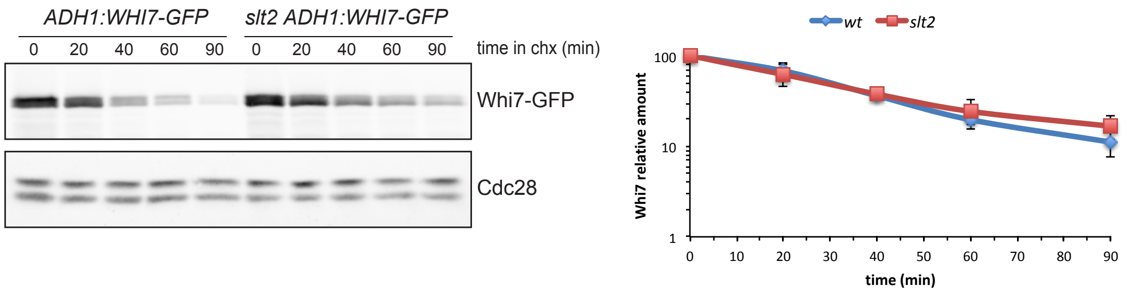

Figure S3. Slt2 inactivation does not affect Whi7 protein stability. Exponentially growing cultures of the wt (W303) and slt2 (JCY2040) strains transformed with the pADH1:WHI7-GFP$_4$ plasmid were incubated in the presence of 100 μg/mL cycloheximide. Whi7 protein level was analyzed at the indicated time after the addition of cycloheximide by Western blot. Cdc28 is shown as loading control. Graph represents the relative amount of Whi7 protein related to Cdc28.

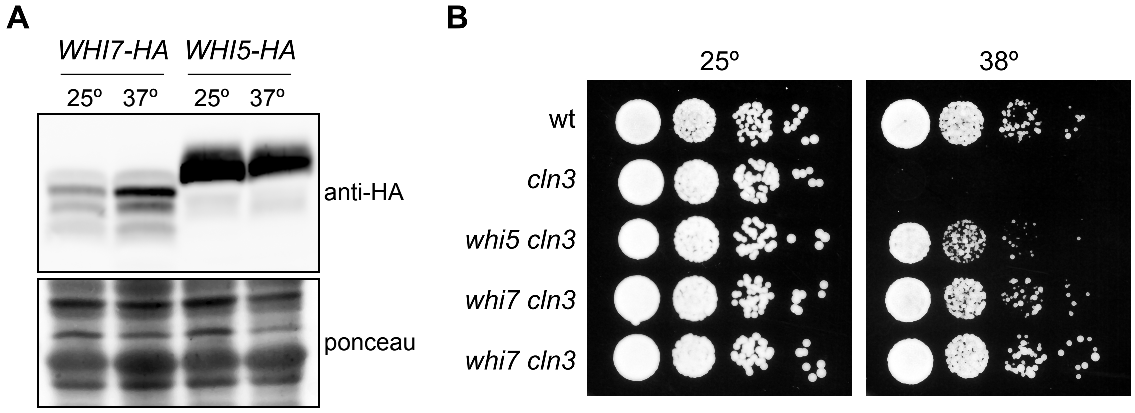

Figure S4. Comparative analysis of Whi7 and Whi5 function under heat-stress conditions. *A)* Exponentially growing cells of *WHI7-HA* (JCY2015) or *WHI5-HA* (JCY2036) strains were incubated at 37°C for three hours. Whi7 and Whi5 protein level was analysed by Western blot. Ponceau staining is shown as loading control. *B)* 10-fold serial dilutions from cultures of wild type (W303), *cln3* (MT244), *whi5 cln3* (JCY1875) and *whi7 cln3* (JCY1868) strains were spotted onto YPD plates and incubated at 25°C or 38°C for 3 days.

Journal of Cell Science • Supplementary information

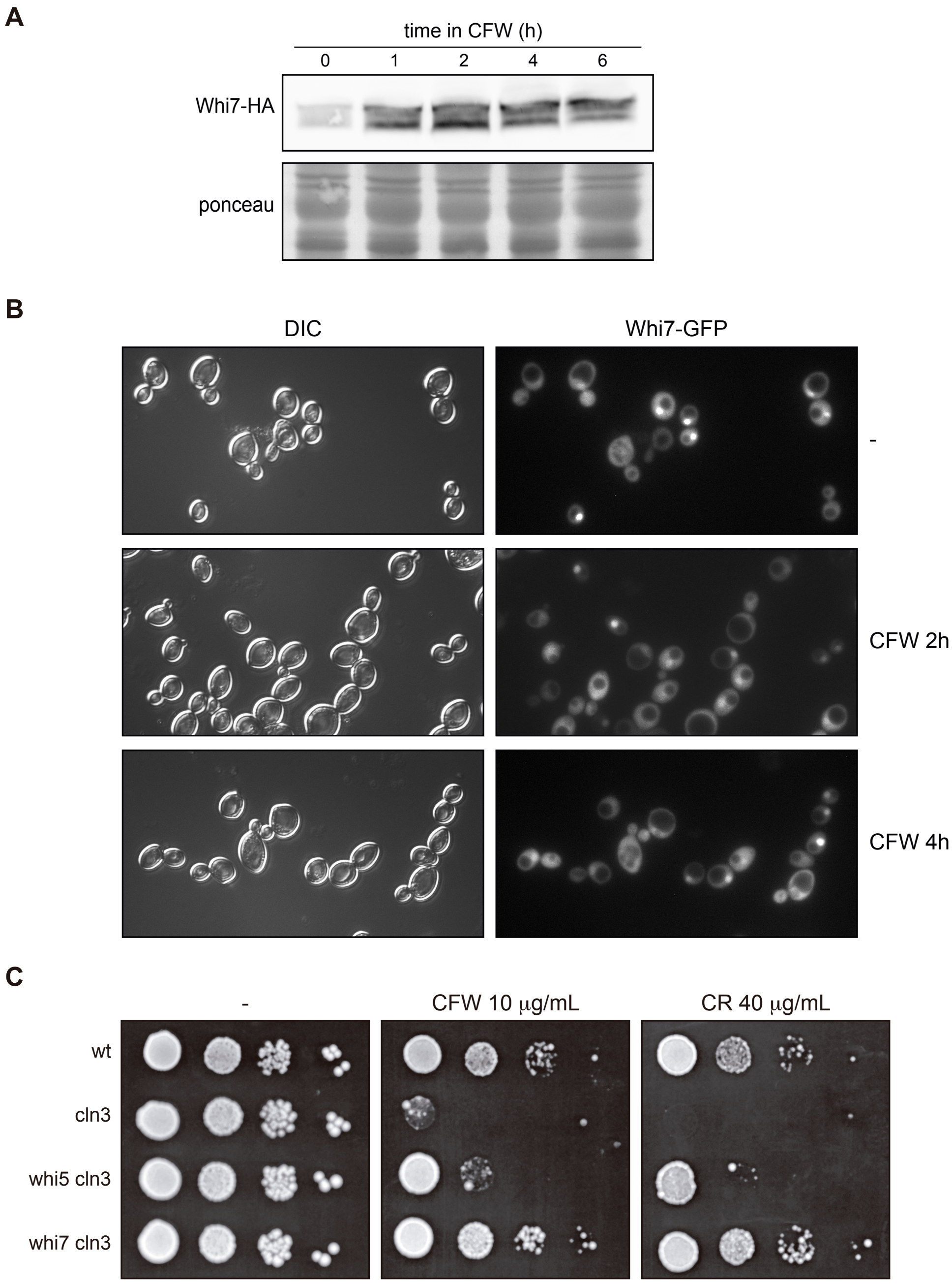

Figure S5. Analysis of Whi7 function in the presence of Calcofluor White. *A)* Exponentially growing cells of the *WHI7-HA* (JCY2015) strain were incubated in the presence of 10 µg/mL Calcofluor White (CFW). Whi7 protein level was analysed by Western blot at the indicated times. The ponceau staining of the membrane is shown as loading control. *B)* Exponentially growing cells of the wild type (W303-1a) strain transformed with plasmid pADH1:WHI7-GFP_4 were analysed by fluorescence microscopy at the indicated times after the addition of 10 µg/mL Calcofluor White. GFP signal and DIC images are shown. *C)* 10-fold serial dilutions from cultures of wild type (W303), *cln3* (MT244), *whi5 cln3* (JCY1875) and *whi7 cln3* (JCY1868) strains were spotted onto YPD plates containing 10 µg/mL Calcofluor White or 40 µg/mL Congo Red and incubated at 25°C for 3 days.

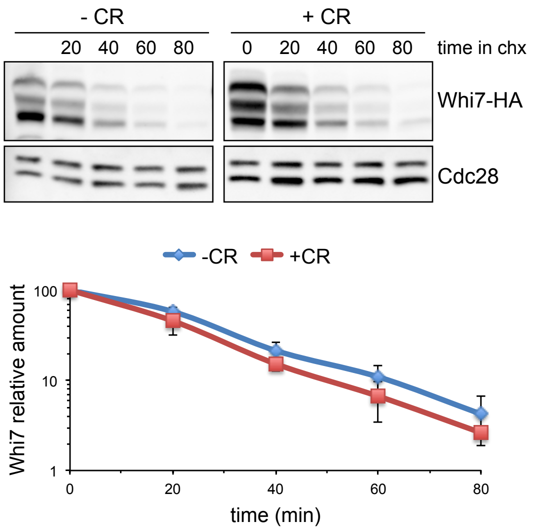

Figure S6. Whi7 stability is not altered in the presence of Congo Red. Exponentially growing cultures of the *WHI7-HA* (JCY1728) strain were split, incubated for four hours in the absence or presence of 40 µg/mL Congo Red and then 100 µg/mL cycloheximide was added. Whi7 protein level was analyzed at the indicated time after the addition of cycloheximide by Western blot. Cdc28 is shown as loading control. Graph represents the relative amount of Whi7 protein related to Cdc28.

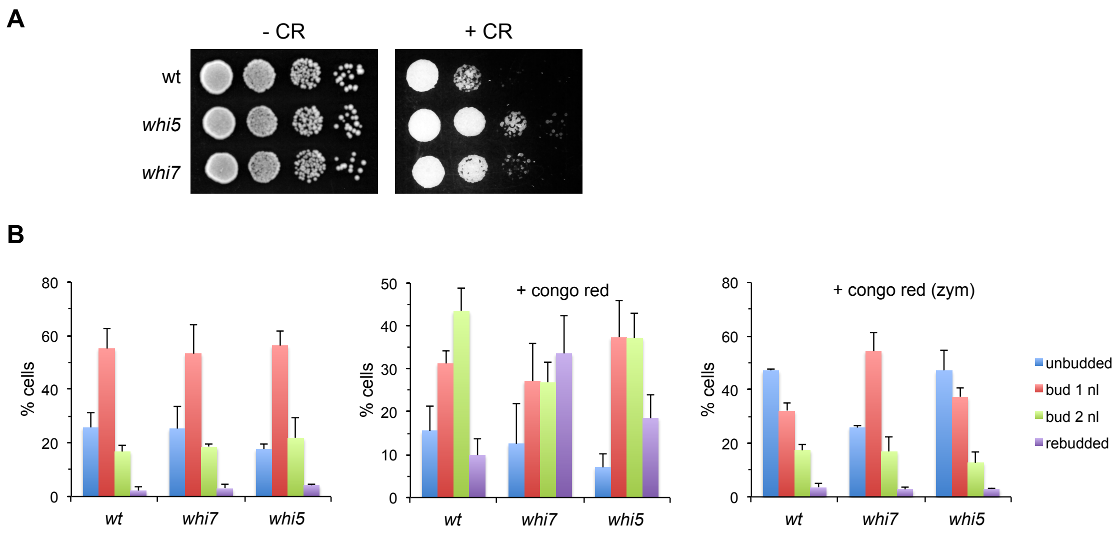

Figure S7. Comparative analysis of Whi7 and Whi5 function under cell wall stress conditions in a wild type background. *A)* 10-fold serial dilutions from cultures of wild type (W303), *whi5* (JCY1784) and *whi7* (JCY1819) strains were spotted onto YPD plates containing or not 40 µg/mL Congo Red and incubated at 25°C for 3 days. *B)* Cell cycle distribution of wild type (W303), *whi5* (JCY1784) and *whi7* (JCY1819) cells after four hours since the addition of Congo Red. Fixed cells were also treated with zymolyase (zym) to digest cell wall. Cells were scored as unbudded, budded with one nucleus (1nl), budded with two nuclei (2nl) or re-budded. Values are the mean and s.d. derived from three experiments.

Journal of Cell Science • Supplementary information

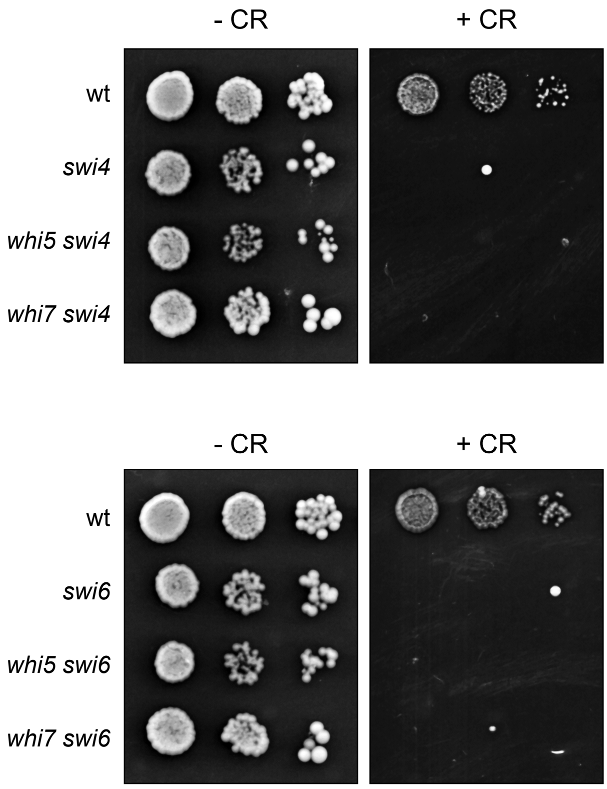

Figure S8. Whi7 function under cell wall stress conditions requires Swi4 and Swi6. 10-fold serial dilutions from cultures of wild type (W303), *swi4* (JCY167), *whi5 swi4* (JCY2237), *whi7 swi4* (JCY1872), *swi6* (JCY221), *whi5 swi6* (JCY2095), *whi7 swi6* (JCY1880) strains were spotted onto YPD plates containing or not 40 µg/mL Congo Red and incubated at 25°C for 3 days.

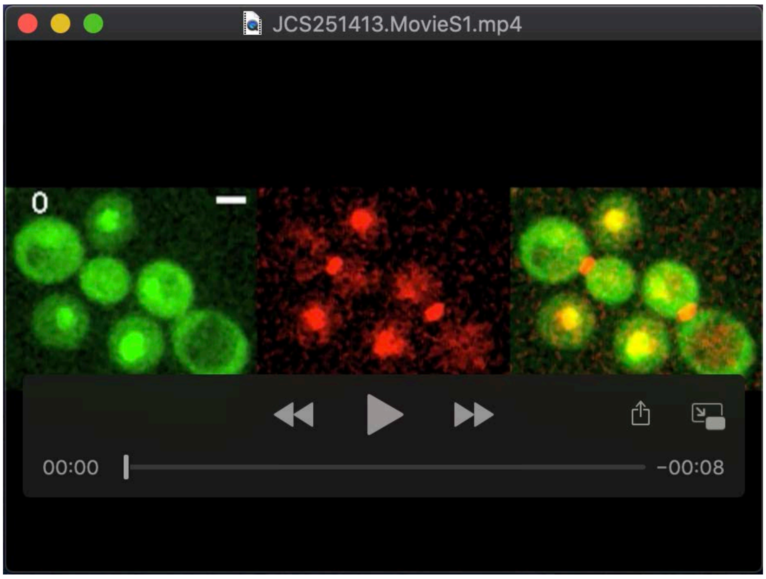

Movie 1. Comparative analysis of the subcellular localization of Whi7 and Whi5 as described in Figure 3C.

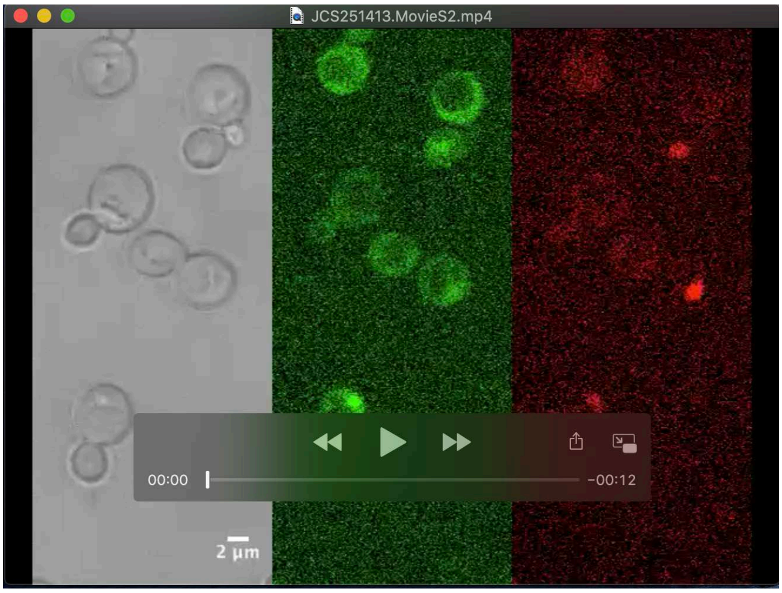

Movie 2. Comparative analysis of the subcellular localization of Whi7 and Whi5 expressed at endogenous level as described in Figure 3D.

Journal of Cell Science • Supplementary information

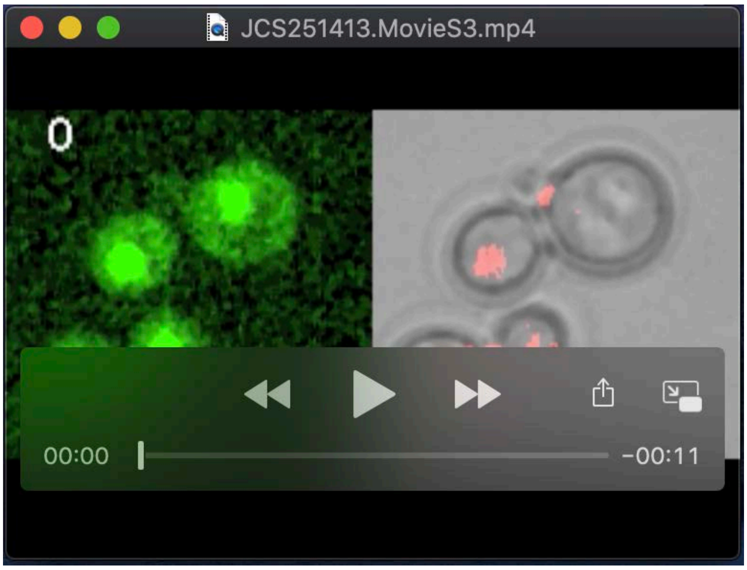

Movie 3. Analysis of the subcellular localization of non-phosphorylatable Whi7 protein as described in Figure 4B.

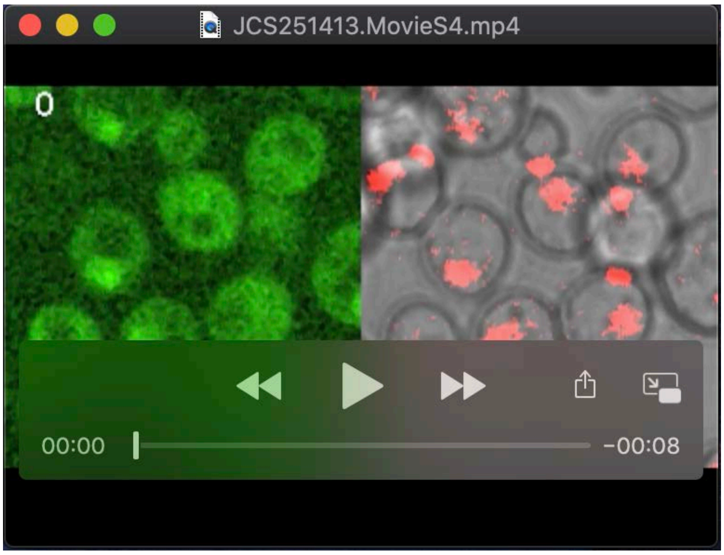

Movie 4. Analysis of Whi7 subcellular localization in a mutant in the Msn5 karyopherin as described in Figure 5C.

Journal of Cell Science • Supplementary information
