# Supplementary material for: The budding yeast Start repressor Whi7 differs in regulation from Whi5, emerging as a major cell cycle brake in response to stress
Source: J Cell Sci. 2020 Dec 21;133(24):jcs251413. doi: 10.1242/jcs.251413 (PMC7774886; doi:10.1242/jcs.251413)
Supplement: Reviewer comments [file joces-133-251413_review_history.pdf]

The budding yeast Start repressor Whi7 differs in regulation from Whi5, emerging as a major cell cycle brake in response to stress

Ester Méndez, Mercè Gomar-Alba, M. Carmen Bañó, Manuel Mendoza, Inma Quilis and J. Carlos Igual
DOI: 10.1242/jcs.251413

Editor: David Glover

Review timeline
Original submission: 8 July 2020
Editorial decision: 29 September 2020
First revision received: 8 October 2020
Accepted: 9 November 2020

Original submission

First decision letter

MS ID#: JOCES/2020/251413

MS TITLE: Start repressor Whi7 is differentially regulated to Whi5 emerging as a major cell cycle brake in response to stress

AUTHORS: Ester Mendez, Merce Gomar-Alba, M. Carmen Bano, Manuel Mendoza, Inma Quilis, and J. Carlos Igual
ARTICLE TYPE: Research Article

We have now reached a decision on the above manuscript.

To see the reviewers' reports and a copy of this decision letter, please go to: https://submit-jcs.biologists.org and click on the 'Manuscripts with Decisions' queue in the Author Area. (Corresponding author only has access to reviews.)

As you will see, the reviewers raise a number of substantial criticisms that prevent me from accepting the paper at this stage. They suggest, however, that a revised version might prove acceptable, if you can address their concerns. If you think that you can deal satisfactorily with the criticisms on revision, I would be pleased to see a revised manuscript. We would then return it to the reviewers.

We are aware that you may currently be unable to access the lab to undertake experimental revisions. If it would be helpful, we encourage you to contact us to discuss your revision in greater detail. Please send us a point-by-point response indicating where you are able to address concerns raised (either experimentally or by changes to the text) and where you will not be able to do so within the normal timeframe of a revision. We will then provide further guidance. Please also note that we are happy to extend revision timeframes as necessary.

Please ensure that you clearly highlight all changes made in the revised manuscript. Please avoid using 'Tracked changes' in Word files as these are lost in PDF conversion.

I should be grateful if you would also provide a point-by-point response detailing how you have dealt with the points raised by the reviewers in the 'Response to Reviewers' box. Please attend to

© 2020. Published by The Company of Biologists under the terms of the Creative Commons Attribution License (https://creativecommons.org/licenses/by/4.0/).

all of the reviewers' comments. If you do not agree with any of their criticisms or suggestions please explain clearly why this is so.

Reviewer 1

Advance summary and potential significance to field

The paper by Mendez et al describes key differences in the properties and physiological relevance of two Start repressors, Whi5 and Whi7, in budding yeast. Interestingly, they clearly demonstrate a specific role for Whi7 in the G1 arrest caused by cell wall stress. In my view, this is a sound experimental work that addresses the issue of functional redundancy/diversity in a key molecular mechanism.

Comments for the author

Nonetheless, there are some questions that need to be clarified and, in a few instances, might require simple experiments with the tools and reagents already developed by the authors.

Major points:
1. It seems that the decrease in WHI7 expression in non-stressed slt2 cells is more important at the protein level (Fig 6), suggesting a role for the PKC pathway in stabilizing (perhaps by phosphorylation) Whi7 under normal conditions. The experiments with pkc(ts) involve heat stress and are not informative, but it would be interesting to repeat experiment in Fig 7H with a less potent promoter.
2. Does cell wall damage modify the nucleus/cytoplasm distribution of Whi7?
3. The mechanism of WHI7 transcriptional dependence on the PKC pathway seems to involve Rlm1. Transcriptomic data in Becerra et al 2011 and Hu et al 2007, and ChIP data in Harbison et al 2004 and Heredia et al 2020 (C albicans) should be discussed.
4. Rapamycin increases WHI7 expression (SPELL). Given the connections between the TOR and the PKC pathways, it would be interesting to add this issue to the Discussion section.

Minor points:
1. Whi7 binds strongly to a subset of SBF-driven genes, do the authors think of specificity determinants?
2. It is assumed that Swi6 prevents Swi4 from DNA-binding autoinhibition.
This might explain why Whi7 binding to SCB promoters is (slightly) decreased in swi6 cells.
3. The authors propose that Whi7 would bind MCB promoters through Mbp1. If available, the authors might consider to add data on the RNR1 promoter in wt vs GALp-SWI4 mbp1 cells.
4. The cln3 mutant is strikingly thermosensitive (Fig S3). Is this a background-dependent phenotype?

Becerra M, Lombardía LJ, Lamas-Maceiras M, Canto E, Rodríguez-Belmonte E Cerdán ME. Comparative transcriptome analysis of yeast strains carrying slt2, rlm1, and pop2 deletions. Genome. 2011 Feb;54(2):99-109.
Harbison CT, Gordon DB, Lee TI, Rinaldi NJ, Macisaac KD, Danford TW, Hannett NM, Tagne JB, Reynolds DB, Yoo J, Jennings EG, Zeitlinger J, Pokholok DK Kellis M, Rolfe PA, Takusagawa KT, Lander ES, Gifford DK, Fraenkel E, Young RA. Transcriptional regulatory code of a eukaryotic genome. Nature. 2004 Sep 2;431(7004):99-104.
Heredia MY, Ikeh MAC, Gunasekaran D, Conrad KA, Filimonava S, Marotta DH Nobile CJ, Rauceo JM. An expanded cell wall damage signaling network is comprised of the transcription factors Rlm1 and Sko1 in Candida albicans.
PLoS Genet. 2020 Jul 8;16(7):e1008908.
Hu Z, Killion PJ, Iyer VR. Genetic reconstruction of a functional transcriptional regulatory network. Nat Genet. 2007 May;39(5):683-7.

© 2020. Published by The Company of Biologists under the terms of the Creative Commons Attribution License (https://creativecommons.org/licenses/by/4.0/).

Reviewer 2

Advance summary and potential significance to field

The transcriptional repressor Whi5 (~mammalian Rb) coordinates START with cell growth & metabolism in budding yeast. Loss of Whi5 activity allows small cells to pass START. In this capacity, Whi5 blocks activity of the SBF complex (Swi4-Swi6) thru recruitment of HDACs, and inactivation of Whi5 via Cdc28/Cln3 phosphorylation initiates progression past START. Whi7 is a paralog of Whi5. It is a Cdc28 target and shares overlapping Whi5 promoter specificities. However, loss of Whi7 does not disrupt cell size control at START, and Whi7 is expressed at much lower levels then Whi5. Interestingly, Whi7 expression is influenced by the cell wall integrity pathway.

Here, the authors carry out a comprehensive comparison of Whi7 and Whi5 and an investigation of the relationship between Whi7 and stress (cell wall integrity / PKC pathway) in G1 and START. Their data clearly show that Whi7 prefers promoters that influence cell wall integrity, and that this preference is influenced by the SBF component Swi4, but interestingly not Swi6. They also show that the nuclear import determinants differ between Whi5 and Whi7. They also further characterize of the relationship between Whi7 expression and the cell wall integrity pathway, and discover that basal expression of Whi7 (but not Whi5) depends on Slt2 and Rlm1, both players in the MAPK response to cell wall stress that is downstream of PKC. Finally, they show that cln3 sensitivity to congo red, a cell wall poison, is due to a Whi7- dependent arrest in G1. As far as I know, this is a novel result that really confirms the role of Whi7 as a mediator between cell wall stress and START. The data presented in the article support these conclusions.

Comments for the author

Minor comments

1) Whi7 has been found to associate with the ER, where it tethers Cdc28/Cln3 prior to START (Yayha et al). In that article, it appeared to me that Whi7 did not localize to the nucleus. I think that it would be helpful if the authors discuss why Whi7 did not appear to localize to the nucleus in that article.

2) In the introduction (lines 150-152), the authors downplay the effects of Whi5 and Whi7 on cell size (…due to differences in cellular levels of unstable Whi7) but seem to highlight the effects in the discussion (Lines 338 to 341). It would help if the authors were more consistent in the introduction and discussion on this matter.

3) Line 416: the authors say that reduced expression of WHI7 in a pkc1 mutant are unpublished data, yet these data are shown in Figure 6A.

4) I think that the y-axes of Figure 2A should be the same (the Whi7 extends to 7, and the Whi5 to 14) because the difference may mislead readers to believe that Whi7 plays a greater role under normal conditions than it does.

5) Figure 2F: it would be helpful if the diagram contained specific information about **SBF targets** and **MBF targets** (what are they, how do they differ).

6) I think that the titles of the figures should capture the main conclusion of each figure. As it stands, the figure legends just describe the analyses, which isn't helpful for readers looking for conclusions / interpretations.

© 2020. Published by The Company of Biologists under the terms of the Creative Commons Attribution License (https://creativecommons.org/licenses/by/4.0/).

First revision

Author response to reviewers' comments

Reviewer 1

Major points

1) We have analysed protein level in *slt2* mutant expressing Whi7 under the control of the *ADH1* promoter. As observed for the *GAL1* promoter, no significant differences between wt and *slt2* mutant were observed. Moreover, we carried out a shut-off experiment, which indicated that Slt2 does not affect Whi7 stability. These results are now included in new Figure S3 and commented in line 273. As Reviewer remarks, drop of Whi7 protein is more drastic than that of *WHI7* mRNA in Fig 6. It could be hypothesized for instance that Slt2 could affect *WHI7* mRNA translation rate. However, we are not sure whether there is necessarily an exact quantitative correlation of the fold change between mRNA and protein level.

2) We have analysed Whi7 localization under cell wall stress and no differences were observed. The result is now included in new Figure S5. Note that the analysis was carried out using cell wall stressor Calcofluor White (CFW). In Fig S5BA and C we show that CFW acts as Congo Red (in fact both compounds interfere with chitin).

3) Genomic approaches showed that *WHI7* expression increased after cell wall damage (*Boorsma et al 2004 Yeast 21:413* and *Lagorce et al. 2003 JBC 278:20345*). Other genomic studies (our unpublished results and *García et al 2004 JBC 279:15183*) directly connect *WHI7* expression to components of the PKC pathway like Pkc1 and Rlm1 respectively. More recently, it was described the binding of Rlm1 to specific sites in *WHI7* promoter in the presence of CR (*Sanz et al. 2012, MBC 23:2805*). These results are commented in the Result section lines 253 and 268 and in the Discussion section line 419.

To our knowledge, there are not genomic studies reporting the regulation of *WHI7* expression by the PKC pathway in normal conditions. We have checked the manuscripts indicated by the Reviewer. Hu et al. carried out an expression analysis in normal conditions in the *rlm1* mutant strain. However, nothing is commented about *WHI7* and in the primary data the *WHI7* value is indicated as 'null'. In Becerra et al., *WHI7* is not included in the Table that show the 'Genes affected by *slt2* or *rlm1* deletion'. Unfortunately, we can get access to the primary data in the address indicated in the manuscript. About genomic ChIP data, we have not look into detail because we prefer the *ad hoc* demonstration described by Sanz et al 2012 (and in other Dr. J. Arroyo's group manuscripts) of Rlm1 binding to *WHI7* promoter.

4) We were aware of the induction of *WHI7* gene expression after treatment with rapamycin (*Hardwick et al 1999 PNAS 96:14866*). However, the connection between Tor and PKC involves the TORC2 complex, whereas rapamycin inhibits the TORC1 complex. This complex is involved in nutrient sensing and growth control and probably the *WHI7* induction by rapamycin is reflecting that Whi7 could be involved in the response to nutritional stress conditions. In fact, *WHI7* is induced by several stresses (*Gasch et al 2000 Mol Biol Cell* ***11****, 4241* and *Waern and Snyder 2013 G3, 3:343*). These observations support a more general role for Whi7 in the response to stress that the specific response to cell wall damage described in this manuscript. Future work of the group is trying to pursue this hypothesis.

In the Introduction section line 141, we commented Whi7 induction by stress and refer to the two manuscripts covering multiple stresses and the specific cell wall damage stress manuscript given the relevance for this work.

Minor points

1) As Reviewer indicates, some specific determinants must explain the different promoter binding preferences of Whi7 and Whi5. In fact, we provide some clues that could help to explain this, as we described the ability of Whi7, but not Whi5, to act through monomeric Swi4 and Mbp1. We have tested a small subset of target genes, but, interestingly, the genes to

© 2020. Published by The Company of Biologists under the terms of the Creative Commons Attribution License (https://creativecommons.org/licenses/by/4.0/).

which Whi7 shows more binding preference than Whi5, have a higher binding of monomeric Swi4 (*FKS1*) or Mbp1 (*FKS1, RNR1*). However, probably it would be necessary a more comprehensive genomic approach to drive definitive conclusions. It cannot be discarded the existence of additional yet unknown differences in the way both proteins associate to promoters.

2) As showed in Fig 2B, binding of Swi4 is importantly affected by Swi6 absence (except in the case of the *FKS1* gene). This could contribute to the decrease in Whi7 association, as Reviewer indicates. However, we previously described that Whi7 associates to SBF. We believe that the decreased binding of Whi7 in the *swi6* mutant could be probably more, or also, related to the absence of SBF.

3) Now we have analysed the samples for the *RNR1* promoter. As it is shown in new Figure 2E, the result confirmed the conclusion that Whi7 association to promoters in the absence of Swi4 is mediated by Mbp1.

4) We have worked with two different *cln3* mutant strains in the W303 background and both of them are lethal at 38º. We do not know whether this could be a specific trait of W303 background or be shared with other genetic backgrounds.

Reviewer 2

1) We don't know the exact reason for this. The low level of Whi7 protein makes challenging address its detection by fluorescence microscopy at endogenous level. Thus, we analysed it in live cells by using the NeonGreen tag, brighter than m-Citrine or GFP. *Yahya et al* used a HA-tagging that involves a more noisy/tricky assay (indirect immunofluorescence in fixed cells). It could be possible that the different approach could affect the result. Maybe the mild conditions used to save peripheral ER structure could make difficult to the antibody to get inside the nucleus. What we have demonstrated in the characterization of Whi7 is that the protein has indeed a nuclear function binding to promoters.

2) We have changed the Introduction sentence to better resemble the Discussion. Now it is stated in line 151-154: '*Effect on cell size indicates that Whi5 has a greater relevance than Whi7 during the Start transition. The differences in cellular levels caused by the high unstability of Whi7 protein (Gomar-Alba et al., 2017) could contribute to explain this; however, we wonder whether, in addition to this quantitative difference, Whi7 and Whi5 could also show qualitative differences.'*

3) This sentence has been deleted.

4) I understand the Reviewer comment, but the idea in this figure is to clearly show the effect of the indicated mutation on the binding of both repressors, and not to directly compare the strength of the binding of both repressors. This can be assessed looking at the axis scale. To use the same scale would unnecessarily minimize the image for the effect of mutation on Whi7 binding.

5) We have indicated now SBF and MBF target genes in Figure 2F.

6) The figure titles have been changed as suggested.

© 2020. Published by The Company of Biologists under the terms of the Creative Commons Attribution License (https://creativecommons.org/licenses/by/4.0/).

Second decision letter

MS ID#: JOCES/2020/251413

MS TITLE: Start repressor Whi7 is differentially regulated to Whi5 emerging as a major cell cycle brake in response to stress

AUTHORS: Ester Mendez, Merce Gomar-Alba, M. Carmen Bano, Manuel Mendoza, Inma Quilis, and J. Carlos Igual
ARTICLE TYPE: Research Article

I am happy to tell you that your manuscript has been accepted for publication in Journal of Cell Science, pending standard ethics checks.

Reviewer 1

Advance summary and potential significance to field

The paper by Mendez et al describes key differences in the properties and physiological relevance of two Start repressors, Whi5 and Whi7, in budding yeast. Interestingly, they clearly demonstrate a specific role for Whi7 in the G1 arrest caused by cell wall stress. In my view, this is a sound experimental work that addresses the issue of functional redundancy/diversity in a key molecular mechanism.

Comments for the author

The authors have perfectly addressed all of my questions.

Reviewer 2

Comments for the author

I am satisfied with the authors' responses to the reviewers and believe that the article is not acceptable for publication in JCS.

© 2020. Published by The Company of Biologists under the terms of the Creative Commons Attribution License (https://creativecommons.org/licenses/by/4.0/).
